# Systematic Optimization of Protein Secretory Pathways in *Saccharomyces cerevisiae* to Increase Expression of Hepatitis B Small Antigen

**DOI:** 10.3389/fmicb.2017.00875

**Published:** 2017-05-16

**Authors:** Jiayuan Sheng, Hunter Flick, Xueyang Feng

**Affiliations:** ^1^Department of Biological Systems Engineering, Virginia Polytechnic Institute and State UniversityBlacksburg, VA, United States; ^2^Department of Chemical Engineering, Virginia Polytechnic Institute and State UniversityBlacksburg, VA, United States

**Keywords:** yeast knockout, HBsAg, protein express pathway, synergy, CRISPR

## Abstract

Hepatitis B is a major disease that chronically infects millions of people in the world, especially in developing countries. Currently, one of the effective vaccines to prevent Hepatitis B is the Hepatitis B Small Antigen (HBsAg), which is mainly produced by the recombinant yeast *Saccharomyces cerevisiae*. In order to bring down the price, which is still too high for people in developing countries to afford, it is important to understand key cellular processes that limit protein expression. In this study, we took advantage of yeast knockout collection (YKO) and screened 194 *S. cerevisiae* strains with single gene knocked out in four major steps of the protein secretory pathway, i.e., endoplasmic-reticulum (ER)-associated protein degradation, protein folding, unfolded protein response (UPR), and translocation and exocytosis. The screening showed that the single deletion of YPT32, SBH1, and HSP42 led to the most significant increase of HBsAg expression over the wild type while the deletion of IRE1 led to a profound decrease of HBsAg expression. The synergistic effects of gene knockout and gene overexpression were next tested. We found that simultaneously deleting YPT32 and overexpressing IRE1 led to a 2.12-fold increase in HBsAg expression over the wild type strain. The results of this study revealed novel genetic targets of protein secretory pathways that could potentially improve the manufacturing of broad scope vaccines in a cost-effective way using recombinant *S. cerevisiae*.

## Introduction

Hepatitis B is an infectious disease caused by the hepatitis B virus (HBV) which affects the liver (Hollinger and Liang, 2001). It causes both acute and chronic infections and may take 30–180 days for symptoms to develop (Hollinger and Liang, 2001). In 1963, researchers discovered “Australia Antigen” (now called HBsAg) in the serum of an Australian Aboriginal person which paved the road to the development of HBV vaccine (Alter and Blumberg, 1966). The first generation of HBV vaccine was derived from blood serum but was withdrawn from the marketplace in 1986 when researchers succeeded in producing the noninfectious surface protein antigen in yeast *Saccharomyces cerevisiae* without danger of introducing actual viral DNA into the final product (Nielsen, 2013). This kind of HBV vaccine is the most widely used in the world today. However, the price for the HBV vaccine is still too high (~$20/dose) for people in developing countries to afford. Improving the recombinant protein expression efficiency could allow companies to lower the prices of HBV vaccine and make it being more accessible throughout the world.

As an organism generally recognized as safe (GRAS) (Mattia and Merker, 2008), yeast strain such as *S. cerevisiae* is more advantageous than bacteria when being used to produce biopharmaceuticals, because it allows proper protein folding, secrets protein to the extracellular medium, and most importantly, performs proper post-translational modifications of the protein (Wildt and Gerngross, 2005; Nielsen, 2013). Because of the low level of protein expression in eukaryotic systems, many attempts have been done to engineer yeast for improved protein production (Palomares et al., 2004), which includes optimization of fermentation process, selection of the expression vectors systems, searching the signal sequence for extracellular targeting, and engineering host strains for better protein folding and post-translational modification (Idiris et al., 2010). However, most of these efforts were usually found to work successfully only for one (or a few) protein(s) which could not be expanded as a general way for the production of a range of different recombinant proteins (Hou et al., 2012a), and the protein yield of yeast could be 100- to 1,000-fold lower than the theoretically estimated range (Robson, 2007). Therefore, integration of genetic engineering with systems biology becomes urgent for improving the recombinant protein expression in yeast.

**Figure 1:**
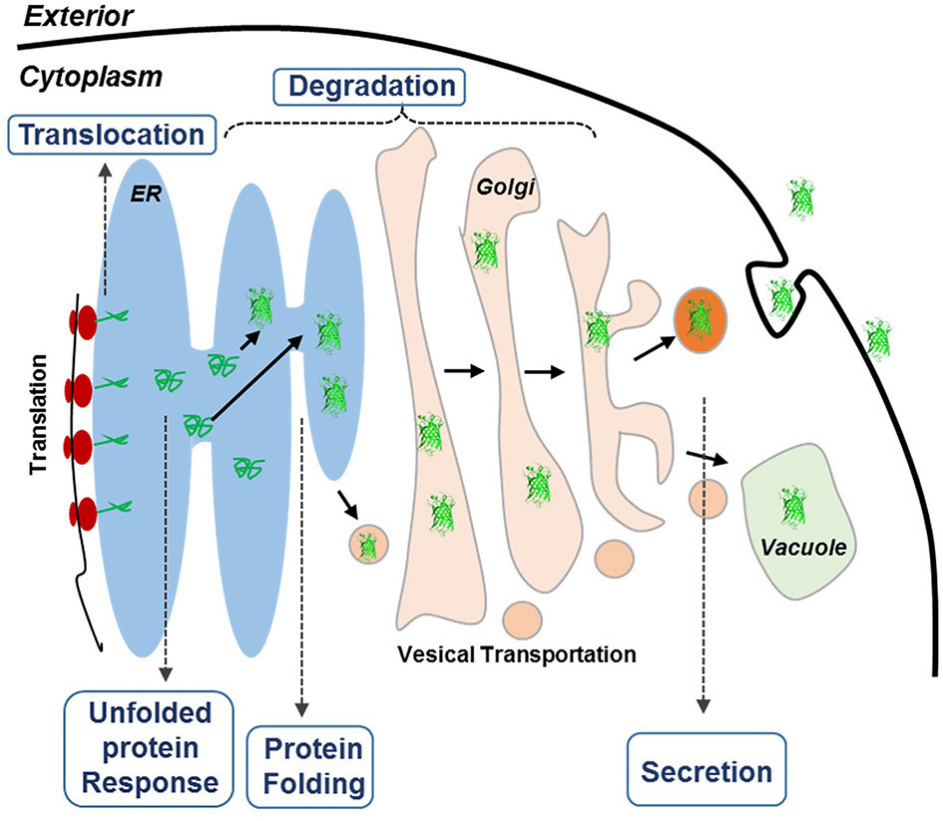
**Schematic overview of the secretory pathways in yeast**. The goal of this research is to improve the expression of HBsAg. Proteins targeted for secretion enter the endoplasmic reticulum (ER). For the correctly folded proteins, they enter the secretory pathway, whereas misfolded proteins cause ER stress, leading to the activation of the unfolded protein response (UPR) that results in activation of a large number of cellular processes. UPR also up-regulates ER-associated degradation (ERAD) where the unfolded proteins are exported from the ER, ubiquitinated and hereby targeted for degradation by the proteasome (ubiquitin-proteasome system, UPS).

Recently, systematic analysis of *Pichia pastoris* by population-based analysis of the genome (De Schutter et al., 2009; Mattanovich et al., 2009; Stadlmayr et al., 2010), transcriptome (Gasser et al., 2007; Resina et al., 2007; Graf et al., 2008), and proteome (Dragosits et al., 2009) has revealed an interesting phenomenon that certain genes in secretory pathway could limit the protein productivity (Love et al., 2012). Another transcriptome analysis on three α-amylase over-producing *Aspergillus oryzae* strains has also identified a complete list of the putative secretome and confirmed its effect on the overproduction of amylase (Liu et al., 2014). Because the protein secretory pathways are conserved in eukaryotic organisms, we hypothesized in this study that genes involved in protein secretory pathways could be rate limiting for protein expression in *S. cerevisiae*. Protein secretory in yeast is a complicate process (Figure 1), involving hundreds of proteins that are responsible for different purposes. In general, proteins start their journey on the intracellular secretory pathway by entering the ER lumen via co-translocation or post-translational translocation, in which the proteins are properly folded under strict quality control (QC) (Dobson, 2004; Anelli and Sitia, 2008). A set of covalent modifications, including signal sequence processing, disulfide bond formation, N-glycosylation, degradation and sorting, are conducted in the ER. Only those proteins that are properly folded and assembled can be exported from the ER to the Golgi apparatus for further modification, followed by being transported to the extracellular space, vacuoles, or other organelles (Klausner, 1989). Meanwhile, the proteins that are misfolded or aggregated in the ER are recognized by the QC system. Through the ER-associated protein degradation (ERAD) (Schroder and Kaufman, 2005), these misfolded proteins are eventually redirected to the cytosol for degradation. In addition, partially misfolded proteins could lead to the induction of unfolded protein response (UPR) (Ferreira et al., 2002), which stimulates proteolysis by ERAD (Gasser et al., 2008). Promoted by current post-genomic systems biology tools, current findings for protein expression and secretion were especially focused on four topics: (1) engineering protein folding and quality control system in the ER, (2) engineering the intracellular protein trafficking pathway, (3) minimizing post-secretory proteolytic degradation, and (4) engineering post-translational glycosylation (required for glycoproteins; Schekman, 2010). However, the impact of individual genes involved in these bioprocesses on protein expression has not yet been fully studied.

In this study, we applied the yeast knockout collection (YKO) and systemically characterized the *S. cerevisiae* strains BY4741 knockout collection library with 194 single genes deleted in the protein secretory pathway. Specifically, we focused on genes related to ER-associated degradation, protein folding, translocation, and UPR because these processes have been indicated previously to affect protein expression in eukaryotic systems (Travers et al., 2000; Ma and Hendershot, 2001; Mattanovich et al., 2004; Mulder et al., 2004; Zhang and Kaufman, 2006; Gasser et al., 2007; Resina et al., 2007; Idiris et al., 2010; Ciplys et al., 2011; Hou et al., 2012a,b). The yeast deletion collections that carries precise start-to-stop deletions of ~6,000 open reading frames (Giaever and Nislow, 2014) has been proved by numerous studies for their applications in a wide array of genome-wide phenotypic assays that aimed to increase our understanding of biological function for individual genes (Winzeler et al., 1999; Giaever et al., 2002; Ghaemmaghami et al., 2003; Krogan et al., 2006). Taking advantage of this tool allows us to explore a holistic picture of the effects of the genetic perturbations on protein expression. In brief, we found that the deletion of YPT32, SBH1, and HSP42 led to the most dramatic increase of HBsAg expression, with 1.92-, 1.66-, and 1.62-fold increases over the wild type *S. cerevisiae* strain, respectively. The deletion of YPT32 together with the overexpression of IRE1 generated synergistic effect, leading to a 2.12-fold increase in HBsAg expression over the wild type strain. In summary, our discoveries revealed several novel genetic targets for improving HBsAg expression and promoted the manufacturing of broad scope vaccines in a cost-effective way by using recombinant *S. cerevisiae*.

## Methods and materials

### Yeast strains, media, and transformation

The yeast strains used in this study were derived from BY4741. The cell cultures were stored in a 15% v/v glycerol solution at −80°C. *E. coli* Top10 strain was used for maintaining and amplifying plasmids, and recombinant strains were cultured at 37°C in Luria-Bertani (LB) broth. Ampicillin at a concentration of 100 μg/mL was added to the LB medium when required. The BY4741 strains were cultured in YPAD medium. Yeast cells were transformed with plasmids using the LiAc/PEG method as described previously (Bergkessel and Guthrie, 2013). For selection of the yeast transformants, a synthetic complete (SC) medium was used, which contains 0.17% yeast nitrogen base, 0.5% ammonium sulfate, and the appropriate amino acid dropout mix (MP Biomedicals, Solon, OH). A single colony was picked and cultured in 5 mL SC media containing 20 g/L glucose. The cells were cultured at 30°C in disposable culture tubes shaken at 250 rpm for 2 days.

### Plasmid construction

The fused protein HBsAg-GFP was codon optimized and synthesized by IDT gBlock (Table S1). A yeast homologous recombination-based method, DNA assembler, was used to construct the recombinant plasmids (Kim et al., 2013). In detail, the TEF1p promoter, TEF1t terminator, and the HBsAg-GFP gene were amplified by primers incorporated with 40 bp homologous arms by PCR. The PCR amplified fragments were co-transformed with the linearized pRS416 plasmid into *S. cerevisiae*, which led to homologous recombination in a single step. The sequences of primers were listed in Table S1. The similar procedure was used to express IRE1, BCK1, SSA4, OPI1, and EPS, which used ENO2p promoter and ENO2t terminator to express the corresponding genes in pRS415 plasmid. The recombinant plasmids constructed in this study were listed in Table S2.

For gene repression using CRISPR and gRNAs, the plasmid used by Farzadfard et al. (2013) (Addgene reference number: 49014) was used as the backbone to insert gRNAs in order to construct the specific gRNAs targeting YPT32, SBH1, and HSP42. The specificity determinant sequence (SDS) for each gRNA was then cloned into the *Hind*III site of these vectors using the same method (Farzadfard et al., 2013). Sequences of the gRNAs used in this study were listed in Table S1. Multiple gRNA expressing plasmids were efficiently constructed by 3A cloning (Shetty et al., 2011) into pRS415 plasmids. In brief, pRS415 plasmid was first linearized by *EcoR*I and *Pst*I. Then, all the gRNAs were amplified by the same forward primer (containing *EcoR*I site and *Xba*I site) and reverse primer (containing *Spe*I site and *Pst*I site), with the first gRNA was digested by *Eco*RI and *Spe*I and the second gRNA was digested by *Xba*I and *Pst*I. These two fragments and the linearized backbone were mixed together and ligated by T4 DNA ligase. After transformation, the correct transformants were identified for the second round assembling to add the third gRNA. Because *Spe*I and *Xba*I produced compatible cohesive ends, ligation of the upstream and downstream parts produced an 8-bp “mixed” or “scar” sequence between the two parts that could not be recognized by either enzyme (Shetty et al., 2011). During the second round assemble, *EcoR*I and *Spe*I were used to digest the plasmids constructed in the first round and *Xba*I and *Pst*I were used to digest the third gRNA. The pRS415 was prepared in the same way, and were mixed together with the three gRNAs and ligated by T4 DNA ligase. After transformation, the correct transformants containing all three gRNAs were identified for transformation. The 27 plasmids with combinations of three gRNAs were listed in Table S2.

### Correlation of protein expression with florescence

Yeast strain harboring the HBsAg-GFP expression plasmid were grown in 25 mL SC media including all appropriate amino acids and 20 g/L glucose, and allowed to grow for 2 days until saturation. Then, 10 mL of the cell cultures were collected. The cell density was tuned to different OD_600_ (0.1, 0.5, 1, 2, 5) with DI water. Two milliliters yeast cells were collected with different OD_600_ and the cells were centrifuged and suspended in 0.5 ml Protein Extraction Buffer (Thermofisher, US). A wild-type yeast strain without the HBsAg-GFP expression plasmid was used as the control to calibrate the autofluorescence of yeast cells. The cells were broken with glass beads. The resulting samples were then analyzed by SDS-PAGE electrophoresis and western blotting.

For western blotting analysis, the target gel was transferred onto a 0.45-μm nitrocellulose (NC) membrane (Pall, USA), which was then incubated with primary mouse anti-GFP antibody (Qiagen, Germany) and HRP-conjugated secondary antibody of goat anti-mouse IgG subsequently. The membrane was then visualized by DAB. The quantities of target protein were measured by Quantity One (Bio-Rad, USA). For the fluorescence measurement, the plate reader with the exciting wavelength at 488 nm and emission wavelength at 509 nm was used to measure the same batch of GFP-tagged HBsAg proteins as that used in western blotting analysis. The western blotting analysis and the fluorescence measurement, as shown in Figure S1, demonstrated high correlation (*R*^2^ = 0.99), which indicated that it was appropriate to use the fluorescence strength to perform the screening of yeast knock out collection.

### Screening of yeast knockout collection

**Figure 2:**
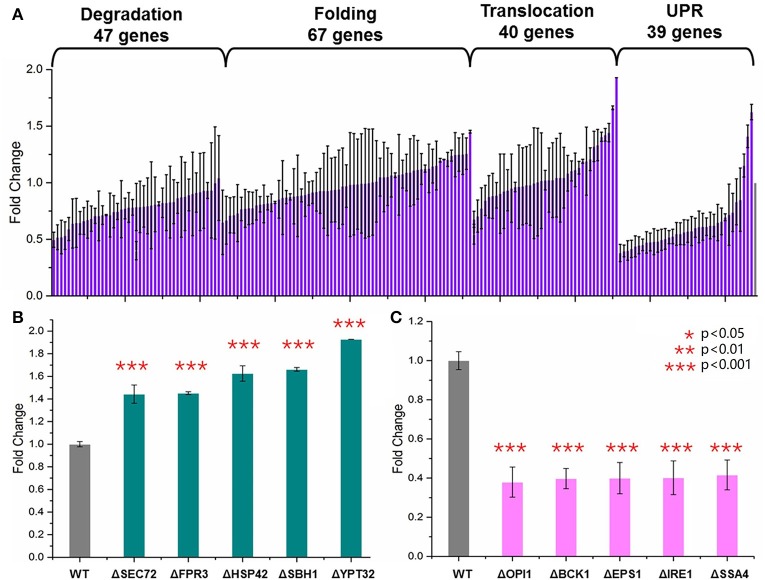
**Screening of the yeast knockout collection. (A)** Overview of the screening results. Totally 194 *S. cerevisiae* strains harboring the HBsAg-GFP plasmid were cultured at 30°C for 48 h in SC medium shaken at 250 rpm. The plate reader was used to measure the OD_600_, and the green florescence with exciting wavelength at 488 nm and the emission wavelength at 509 nm. Each data point represented the mean of biological triplicates and the fluorescence was normalized by OD_600_. The error bars corresponded to standard deviation. Student's *t*-test was used to calculate the *p*-values and we considered two samples were statistically different if *p* < 0.05. **(B)** The five knockout strains leading to the highest HBsAg expression. **(C)** The five knockout strains leading to the lowest HBsAg expression. The *p* < 0.001 was marked with three asterisks; *p* < 0.01 was marked with two asterisks; and *p* < 0.05 was marked with one asterisk.

**Figure 3:**
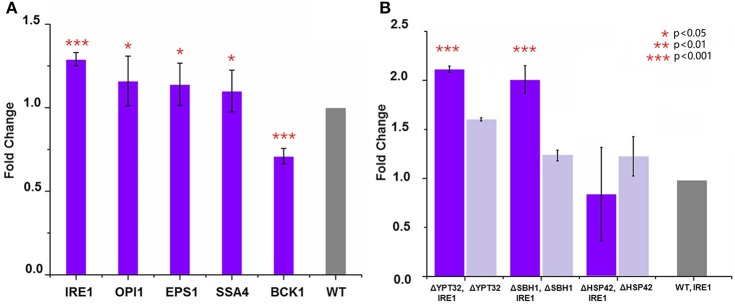
**(A)** Overexpression of genes that led to the lowest HBsAg expression and evaluation of their impacts on HBsAg expression. The wild-type BY4741 strain was co-transformed with the HBsAg-GFP plasmid and the plasmid harboring the target gene (IRE1, OPI1, EPS1, BCK1, or SSA4). Each data point represented the mean of biological triplicates and the fluorescence was normalized by OD_600_. The error bars corresponded to standard deviation. Student's *t*-test was used to calculate the *p*-values and we considered two samples were statistically different if *p* < 0.05. **(B)** Synergistic effects of gene knockout and gene overexpression on HBsAg expression. The yeast knockout strains ΔHSP42, ΔSBH1, and ΔYPT32 were co-transformed with the HBsAg-GFP plasmid and the IRE1 overexpression plasmid. The yeast knockout strains ΔHSP42, ΔSBH1, and ΔYPT32 that were transformed with the HBsAg-GFP plasmid were used as control strains. Significance was calculated by comparing the knockout strains that also overexpressed IRE1 with ones without IRE1 overexpression. All the data was refactored to the wild type strain as fold change. The *p* < 0.001 was marked with three asterisks; *p* < 0.01 was marked with two asterisks; and *p* < 0.05 was marked with one asterisk.

**Figure 4:**
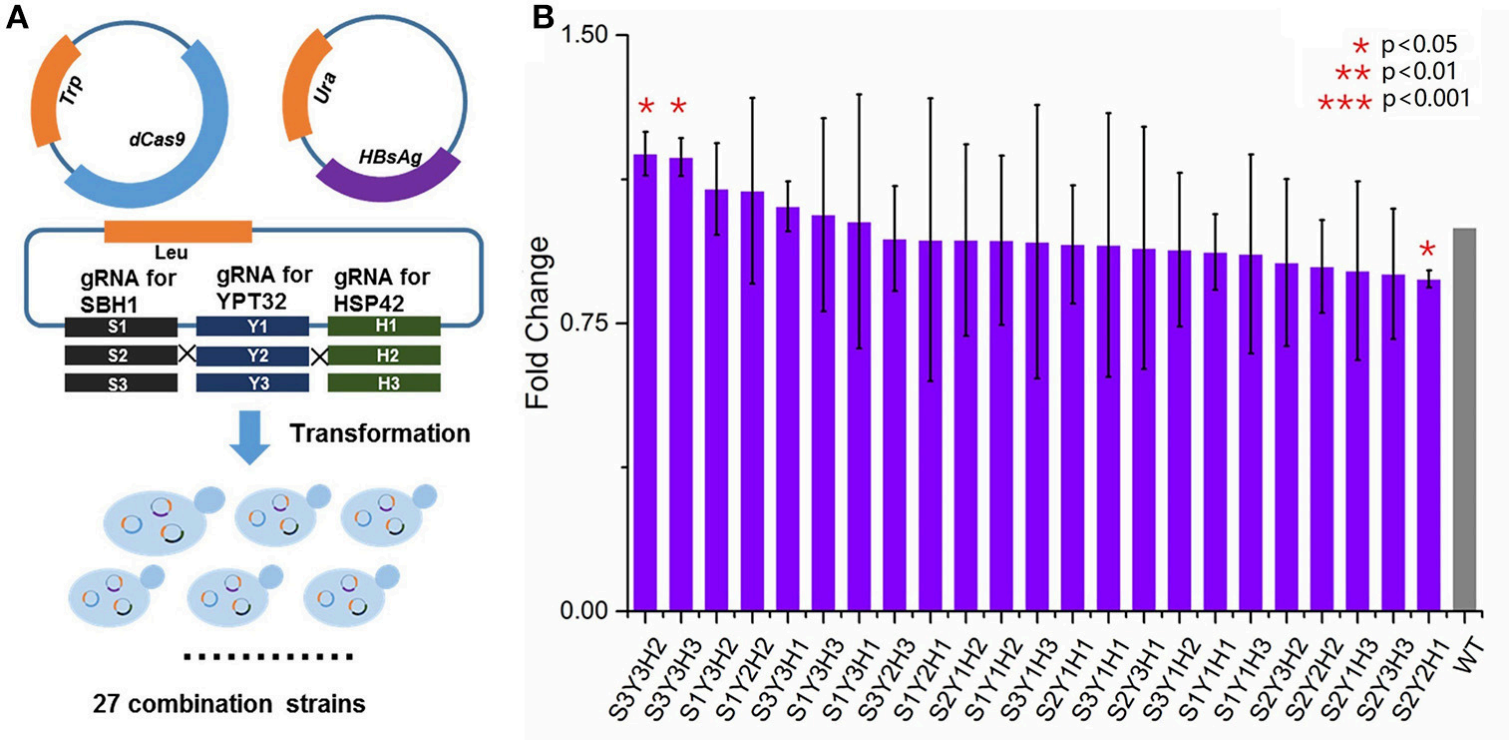
**The impact of multiplex repression of SBH1, HSP42, and YPT32 on HBsAg expression. (A)** The schematic design of multiplex repression of genes by dCas9-based CRISPR system. **(B)** The effects of multiplex gene repression on HBsAg expression. Each data point represented the mean of biological triplicates and the fluorescence was normalized by OD_600_. The error bars corresponded to standard deviation. Student's *t*-test was used to calculate the *p*-values and we considered two samples were statistically different if *p* < 0.05. The *p* < 0.001 was marked with three asterisks; *p* < 0.01 was marked with two asterisks; and *p* < 0.05 was marked with one asterisk. No significant difference between the test strains and the control strain was found in this study. The details of each strain were shown in Table S2.

Yeast knockout collections (BY4741, MATa; his3Δ1; leu2Δ0; met15Δ0; ura3Δ0) were provided commercially as frozen stocks in 96-well plates containing YPD with 15% glycerol (GE Dharmacon, USA). One hundred and ninety-four single gene knockout *S. cerevisiae* strains were selected from this collection. These genes were involved in four major steps of the secretory pathway (Table S3): endoplasmic-reticulum (ER)-associated protein degradation (47 genes), protein folding (67 genes), UPR (39 genes), and translocation and exocytosis (40 genes). For each of the 194 *S. cerevisiae* strains, the HBsAg-GFP plasmid was transformed into these strains using the LiAc/PEG method. An empty HBsAg-GFP backbone plasmid was transformed into the wild type BY4741 as the control strain. After incubation at 30°C for 48 h, three colonies were selected and transformed into SC medium. The cells were cultured at 30°C in disposable culture tubes shaken at 250 rpm for 2 days. Plate reader was used to measure the OD_600_ and the green florescence with exciting wavelength at 488 nm and the emission wavelength at 509 nm. As shown in Figures 2–**4**, each data point represented the mean of biological triplicates and the fluorescence was normalized by OD_600_. The error bars corresponded to standard deviation. Student's *t*-test was used to calculate the *p*-values and we considered two samples were statistically different if *p* < 0.05.

### Creating CRISPRi library for evaluating synergistic effects of multiplex gene repression

Multiplex gene suppression was assayed by creating a library of 27 *S. cerevisiae* strains. Each strain contained (1) a dCas9 expression plasmid, (2) a plasmid containing three gRNAs targeting to three genes that needed to be suppressed, and (3) the HBsAg-GFP expression plasmid. The strain with dCas9 plasmid, HBsAg-GFP plasmid, and an empty gRNA backbone plasmid was used as the control strain (Table S2). All strains were cultured in disposable culture tubes for 2 days at 30°C and shaken at 250 rpm with propitiate SC medium. The plate reader was then used to measure the OD_600_ and the green fluorescence with the exciting wavelength at 488 nm and the emission wavelength at 509 nm. The fluorescence data was normalized by OD_600_. The similar statistical approaches as mentioned previously were used to calculate mean, standard deviation, and *p*-values.

## Results

### Systematic evaluation of the effects of gene knockout on HBsAg expression

GFP is now widely used as a genetic marker because of its unique properties such as high stability, minimal toxicity, non-invasive detection, and the ability to generate the green light without addition of external cofactors (Chalfie et al., 1994; Rosochacki and Matejczyk, 2002). In this study, we fused GFP to the C-terminal of the HBsAg protein in order to establish a fast and high-throughput screening method. We hypothesized that GFP fluorescence of the yeast cells harboring the fused HBsAg-GFP protein could indicate the overall expression level of HBsAg. To validate this hypothesis, we designed experiments to identify the correlation between protein expression characterized by western blotting and the corresponding GFP fluorescence. As shown in Figure S1, the fluorescence strengths and the western blotting measurements of HBsAg-GFP proteins correlated well (*R*^2^ = 0.99), which confirmed that it is appropriate to use the fluorescence strength to perform the screening of yeast knockout strains. It is also worth noticing that the autofluorescence of yeast cells, although existing, was found to be ignorable in the correlation experiment. We next used this screening method to characterize a library of 194 strains from YKOs. Each of the strains had single gene deleted and harbored the HBsAg-GFP expression plasmid (Figure 2A, Table S4). Therefore, by measuring the fluorescence that was normalized by OD_600_ in this study, the effects of single gene deletion on HBsAg expression could be revealed in a high-throughput manner. Overall, we found that the effects of single gene deletions on HBsAg expression were diverse: about 3.2% gene deletions led to decreased HBsAg expression (*p* < 0.05), 91.5% gene deletions did not have significant impact on HBsAg expression (*p* > 0.05), and 5.3% gene deletions led to increased HBsAg expression (*p* < 0.05).

We first identified the five knockout strains that led to the highest improvement of HBsAg expression compared to the wild-type strain: ΔSEC72 (1.44-fold), ΔFPR3 (1.45-fold), ΔHSP42 (1.63-fold), ΔSBH1 (1.66-fold), and ΔYPT32 (1.93-fold) (Figure 2B). Among these five genes, YPT32, SEC72, and SBH1 were involved in the process of protein transport (GO0015031: protein transport). In brief, YPT32 mediates intra-Golgi traffic or the budding of post-Golgi vesicles (Benli et al., 1996). SEC72 is a non-essential subunit of Sec63 complex (Young et al., 2001) and SBH1 is the beta subunit of Sec61p (Soromani et al., 2012). Sec63 complex and Sec61 complex, together with Kar2p/BiP and Lhs1p, form a channel for importing proteins that are SRP-dependent and post-translational SRP-independent into the ER (Young et al., 2001). For the rest two genes (i.e., HSP42 and FPR3), HSP42 plays an important role in UPR (GO:0006950) while FPR3 is found to be important in the protein folding process (GO:0006457). In general, HSP42 has function in both unstressed and stressed cells which could bind and prevent unfolded substrate proteins from irreversibly forming large protein aggregates (Haslbeck et al., 2004). FPR3 is a kind of nucleolar peptidyl-prolyl cis-trans isomerases (PPIase), which affects expression of multiple genes with the PPIase domain acting as a transcriptional repressor when tethered to DNA by lexA (Park et al., 2014). It is beyond the scope of this study to reveal the detailed mechanism of how these gene deletions led to improvement of HBsAg expression. However, the results from our screening, for the first time to our best knowledge, uncovered that knocking out certain genes in protein secretion pathways could improve expression of the recombinant protein. Specifically, three gene deletions, HSP42, SBH1, and YPT32, improved HBsAg expression by more than 1.5-fold, indicating the protein transport could be a crucial step in controlling protein expression in yeast.

We also identified the five knockout strains that led to the most significant decrease of HBsAg expression compared to the wild-type strain: ΔOPI1 (0.379-fold), ΔBCK1 (0.397-fold), ΔEPS1 (0.399-fold), ΔIRE1 (0.402-fold), and ΔSSA4 (0.416-fold) (Figure 2C). Among these five genes, OPI1, BCK1, and IRE1 are involved in endoplasmic reticulum UPR (GO:0030968) while SSA4 and EPS1 are involved in protein folding (GO:0006457). In brief, BCK1 is a mitogen-activated protein kinase kinase kinase (MAPKKK or MEKK), which plays an important role in maintaining cell-wall integrity and preventing fungal cell lysis (Heinisch et al., 1999). OPI1 is a negative regulator of the transcriptional complex INO2-INO4 in response to phospholipid precursor availability (Wagner et al., 2001). IRE1 is a transmembrane protein (Lee et al., 2002) that mediates the UPR by regulating Hac1p synthesis through HAC1 mRNA splicing. For SSA4 and EPS1, EPS1 helps to recognize proteins targeted for ER-associated degradation (ERAD) (Wang and Chang, 1999) while SSA4 is a heat shock protein and is highly induced upon stress with a role in SRP-dependent co-translational protein-membrane targeting and translocation (Boorstein and Craig, 1990). It is worth noticing that all these five genes, along with the HSP42 that was identified as the knockout target, are associated with the UPR.

### Synergistic effect of gene overexpression and gene knockout on HBsAg expression

The fact that deletion of five genes, i.e., ΔOPI1, ΔBCK1, ΔEPS1, ΔIRE1, and ΔSSA4, led to decreased expression of HBsAg indicated a positive role these genes could play in improving HBsAg expression. Therefore, we next tested if overexpression of the five genes (OPI1, BCK1, EPS1, IRE1, and SSA4) individually could improve HBsAg production. In general, we constructed five plasmids (pOPI1, pBCK1, pRSEPS1, pRSIRE1, and pSSA4) using the pRS415 plasmid as the backbone to overexpress each individual gene (Table S2). These plasmids were co-transformed into a wild type BY4741 strain together with the HBsAg expression plasmid (i.e., pHBV-EGFP). We also co-transformed an empty pRS415 with the HBsAg expression plasmid in the wild type BY4741 strain and used it as the control strain. As shown in Figure 3A, the results indicated that the overexpression of IRE1, OPI1, EPS1, and SSA4, respectively, could indeed increase HBsAg expression at different levels (1.3-fold for IRE1, 1.09-fold for OPI1, 1.14-fold for EPS1, and 1.16-fold for SSA4), compared to the control strain. However, the overexpression of BCK1 did not improve HBsAg expression, leading to 0.7-fold change compared to the control strain. To sum, in addition to identifying gene deletions that led to improved HBsAg expression, we also, for the first time to our best knowledge, identified that overexpression of four genes in protein secretory pathway, especially IRE1, led to improved expression of recombinant protein in yeast.

Encouraged by the success of using gene deletion and gene overexpression strategies to improve HBsAg expression in yeast, we next studied if the synergistic effect could be achieved between these strategies. In this study, we chose IRE1 as the target for overexpression and YPT32, SBH1, HSP42 as targets for deletion. We overexpressed IRE1 in the ΔYPT32, ΔSBH1, ΔHSP42 strains, respectively, together with the expression of HBsAg-GFP. It was found that the combination of IRE1 overexpression and the ΔYPT32 further improved HBsAg-GFP production, leading to a 2.12-fold increase compared to the wild type strain that only expressed HBsAg and a 1.31-fold increase compared to ΔYPT32 that only expressed HBsAg (Figure 3B). IRE1 overexpression and the ΔSBH1 also achieved synergy for expressing HBsAg. Nevertheless, combining overexpression of IRE1 and ΔHSP42 failed to increase the HBsAg expression, by so far unknown reasons. The mechanism behind the synergy of gene overexpression and gene knockout is beyond the scope of this study. We are currently designing new experiments to uncover this biomolecular mechanism.

### Synergistic effect of multiplex gene repression on HBsAg expression

We next sought to evaluate the synergistic effect of multiplex gene repression (YPT32, SBH1, and HSP42) on HBsAg expression. This task was done by a CRISPRi system that was previously developed (Larson et al., 2013), which is able to simultaneously target on multiple genes and block the gene expressions. As shown in Figure 4A, in the wild type BY4741 strain, in addition to express HBsAg-GFP, we also expressed a dCas9 protein as well as three guide RNAs that were targeted on different locations of YPT32, SBH1, and HSP42, respectively. We carefully selected the guide RNAs for this CRISPRi system so that the guide RNAs were deployed in three different regions of each gene (i.e., promoter region, the transcription initiation region, and the ORF region) to trigger gene expression at different levels. The efficiency of the individual designed gRNAs was confirmed by qRCR (Figure S2) and the gene repression ranged from 0.7- to 2.7-fold. We next created a gene repression library of 27 strains (three guide RNAs for each of the three regions of the three genes: 3 × 3 × 3 = 27). Of the 27 strains, 23 were successfully developed while 4 strains failed to grow, possibly because of potential off-target effects of CRISPRi system (Larson et al., 2013; Qi et al., 2013; Cho et al., 2014). The wild-type BY4741 strain containing HBsAg-GFP plasmid, the dCas9 plasmid and an empty gRNA backbone plasmid was used as the control strain. We then evaluated the HBsAg expression in these 24 strains. It was found that no significant improvement (*p* > 0.05) was achieved for HBsAg expression (Figure 4B) in any of the 23 strains compared to that of the control strain. This could be attributed to the residue gene expressions because unlike the wild-type CRISPR-Cas9 system, the CRISPRi system could only decrease the gene expression by up to 10- to 300-fold (Qi et al., 2013) instead of fully knocking out the target gene. To test this hypothesis, we tried to create a triple knockout strain, i.e., ΔSBH1ΔYPT32ΔHSP42. We used a protocol developed by Jay Keasling's group (Jakočiūnas et al., 2015) and successfully developed three double knockout strains (Figure S3), i.e., ΔSBH1ΔYPT32, ΔYPT32 ΔHSP42, and ΔHSP42ΔSBH1. However, when we attempted to use the same protocol for developing the triple knockout strain, no colony was found. It is possible that deleting all three genes could disrupt yeast metabolism so much that the cell growth was inhibited (e.g., synthetic lethal).

## Discussion

The hypothesis of this work is that the genes involved in secretory pathways could affect protein expression. This topic is important for producing therapeutic proteins but has not yet been fully studied. We have carefully searched the journals and databases about the effects of secretory pathway genes on protein expression. However, very few papers reported the effects of these genes on recombinant protein expression. This could be due to the general belief that the genes involved in protein secretory pathways only affect protein secretion and their potential effects on protein expression are ignored. As a novel discovery in this study, we found that several genes, such as IRE1, could indeed affect the expression of HBsAg, which validated our hypothesis. Uncovering the biomolecular mechanism of such phenomenon is interesting but beyond the scope of this study. However, we do want to raise a few hypotheses for discussion. For example, intracellular protein concentration could be a possible factor that affects HBsAg expression. It was reported that, in some cases, secretory proteins are retained intracellularly without complete secretion, even though they have entered the ER lumen and folded into their native structure (Idiris et al., 2010). As a result, the ER could be loaded with extremely high concentration of proteins (>100 mg/ml) (Stevens and Argon, 1999; Dimcheff et al., 2004; Anelli and Sitia, 2008), which could trigger stress responses. Interestingly, as found in previous reports (Dimcheff et al., 2004; Ye et al., 2011; Iwata and Koizumi, 2012), a variety of external stimuli (a biotic and biotic stress) such as pathogen invasion, chemical insult have been shown to impose stress on the ER, which leads to alterations of cellular redox equilibrium, disturbances of calcium homeostasis, failure of post-translational modifications, and a general increase in protein synthesis. Therefore, the perturbation of genes involved in protein secretory pathways could induce a similar stress response that led to increase of protein expression. This is possible because perturbation of ER homeostasis causes unfolded proteins to accumulate in the lumen of the ER, triggering an evolutionarily conserved cytoprotective signaling pathway (Zhang and Kaufman, 2006; Ron and Walter, 2007; Urade, 2007; Zhang and Wang, 2012; Lazar et al., 2014). Specifically, it has been reported that IRE1 serves as the ER stress sensor and cell fate executor (Chen and Brandizzi, 2013). It is plausible that the overexpression of IRE1, as discovered in this study, could enhance the stress responses and thus improve HBsAg expression.

Overall, the novelty of this study is two-fold. First, while lots of attempts (Jones, 1991; Robinson et al., 1994; Harmsen et al., 1996; Gleeson et al., 1998; Shusta et al., 1998; Zhang et al., 2001; Ahn et al., 2004; Payne et al., 2008) have been done on evaluating the effects of perturbing secretory pathways on protein secretion, this study was the first report on the effects of perturbing secretory pathways on protein expression. Second, we confirmed that integrating YKOs, genome editing (i.e., CRISPRi method for multiplex gene expression) with high-throughput screening method (i.e., using GFP to characterize protein expression) could lead to system-level discovery of novel strategies for improving protein expression such as synergy of gene knockout and gene overexpression on HBsAg expression.

## Conclusion

To conclude, we found that among the 194 genes tested in this study, the effects of genetic perturbations on HBsAg expression were diverse. We discovered that IRE1 gene played an important role on the expression of HBsAg in *S. cerevisiae*. When overexpressing IRE1 gene in the ΔYPT32 stain, a 2.12-fold increased expression of HBsAg over the wild type was observed. The improvement of HBsAg expression proves that the systemic optimization of protein secretory pathway is crucial for yeast-based vaccine production. To our best knowledge, it is the first time that the effects of genes involved in protein secretory pathways were systemically evaluated on therapeutic protein expression. Because the protein secretion pathways are highly conserved among different eukaryotic systems, it is highly possible that we could extend the findings from this study to other therapeutic proteins.

## Author contributions

JS and XF designed the experiments. JS and HF developed the strains and conducted the experiments. JS, HF, and XF analyzed the data and wrote the manuscript. All authors read and approved the final manuscript.

### Conflict of interest statement

The authors declare that the research was conducted in the absence of any commercial or financial relationships that could be construed as a potential conflict of interest.

## Supplementary material

Table S1 **Sequences of the constructs and primers used in this study**.

Table S2 **The plasmids constructed in this study**.

Table S3 **Genes used for systematic investigation of protein secretory pathway in ***S. cerevisiae*****.

Table S4 **HBsAg expression in 194 yeast knockout strains (same as Figure 2A)**.

Figure S1 **The correlation of the HBsAg-GFP protein expression level and the GFP fluorescence read by plate reader. (A)** The western blotting of HBsAg-GFP proteins form culture with different OD_600_ and the corresponding fluorescence read by plate reader. Western blotting was repeated for three times. M, marker; NC, negative control; BY4741 HBS-GFP: BY4741 strain harboring the HBsAg-GFP expression plasmid; BY4741 Wild Type: a wild-type BY4741 strain without the HBsAg-GFP expression plasmid, which was used as the control to calibrate the autofluorescence of yeast cells. **(B)** Linear correlation between the western blotting reading and the GFP fluorescence (*R*^2^ = 0.99). The autofluorescence of yeast cells, although existing, was found to be ignorable in the correlation experiment. The protein band intensity was calculated by Quantity One software (Bio-Rad, USA).

Figure S2 **qPCR analysis of gene repression by CRISPRi system**. The strains harboring the dCas9 plasmid and the plasmid containing individual gRNAs (e.g., YPT32 site 1) were grown in 25 mL SC media with all appropriate amino acids and 20 g/L glucose for 2 days. The strain harboring the dCas9 plasmid and an empty backbone plasmid for gRNAs was used as the control strain. To analyze gene expression of YPT32, SBH1, and HSP42, 1 ml of the cells were collected and the mRNA was extracted by yeast RNA extraction kit (Thermofisher, USA). Taqman primers designed for YPT32, SBH1, and HSP42 (Thermofisher, USA) were ordered from IDTDNA Inc. to amplify the target genes. ALG9 was used as the housekeeping gene during this assay. The Cq value (quantification cycle) for each sample was refactored into log_2_ (Fold Change) compared with the control strain. Each data point represented the mean of biological triplicates and the error bar was the standard deviation of log_2_ (Fold Change).

Figure S3 **Construction of double- and triple- gene knockout strains**. The protocol developed by Jay Keasling's group (Jakočiūnas et al., 2015) was used to construct the double- and triple- knockout strains. In brief, the wild-type Cas9 expression plasmid was purchased from Addgene (Plasmid #43804) and was transformed into three single knockout strains (i.e., ΔYPT32, ΔSBH1, and ΔHSP42) by using the lithium acetate transformation method. Appropriate gRNA plasmids (i.e., gRNAYPT32 to ΔSBH1 strain; gRNAHSP42 to ΔYTP32 strain and gRNASBH1 to ΔHSP42 strain) were used to guide the Cas9 protein to delete the target genes. One hundred nano grams of gRNA expression plasmid and 1 ng of 90-bp long dsOligos (introducing a stop codon during the DNA homologous repairing) were used per transformation. Yeast cells were then incubated on the galactose SC-medium plates for 3 days to generate the double-knockout strains: ΔSBH1ΔYPT32, ΔYPT32 ΔHSP42, ΔHSP42ΔSBH1. These double-knockout strains were then used as the parent strains to construct triple-knock strain: ΔSBH1ΔYPT32ΔHSP42. However, no colony was found for the triple-knock strain.

## References

[B1] Ahn J. O. Choi E. S. Lee H. W. Hwang S. H. Kim C. S. Jang H. W.. (2004). Enhanced secretion of Bacillus stearothermophilus L1 lipase in *Saccharomyces cerevisiae* by translational fusion to cellulose-binding domain. Appl. Microbiol. Biotechnol. 64, 833–839. 10.1007/s00253-003-1547-5 14740195

[B2] Alter H. J. Blumberg B. S. (1966). Further studies on a “new” human isoprecipitin system (Australia antigen). Blood 27, 297–309. 5930797

[B3] Anelli T. Sitia R. (2008). Protein quality control in the early secretory pathway. EMBO J. 27, 315–327. 10.1038/sj.emboj.7601974 18216874 PMC2234347

[B4] Benli M. Doring F. Robinson D. G. Yang X. Gallwitz D. (1996). Two GTPase isoforms, Ypt31p and Ypt32p, are essential for Golgi function in yeast. EMBO J. 15, 6460–6475. 8978673 PMC452471

[B5] Bergkessel M. Guthrie C. (2013). Chemical transformation of yeast. Methods Enzymol. 529, 311–320. 10.1016/B978-0-12-418687-3.00026-4 24011057

[B6] Boorstein W. R. Craig E. A. (1990). Structure and regulation of the SSA4 HSP70 gene of *Saccharomyces cerevisiae*. J. Biol. Chem. 265, 18912–18921. 2121731

[B7] Chalfie M. Tu Y. Euskirchen G. Ward W. Prasher D. (1994). Green fluorescent protein as a marker for gene expression. Science 263, 802–805. 10.1126/science.8303295 8303295

[B8] Chen Y. Brandizzi F. (2013). IRE1: ER stress sensor and cell fate executor. Trends Cell Biol. 23, 547–555. 10.1016/j.tcb.2013.06.005 23880584 PMC3818365

[B9] Cho S. W. Kim S. Kim Y. Kweon J. Kim H. S. Bae S.. (2014). Analysis of off-target effects of CRISPR/Cas-derived RNA-guided endonucleases and nickases. Genome Res. 24, 132–141. 10.1101/gr.162339.113 24253446 PMC3875854

[B10] Ciplys E. Samuel D. Juozapaitis M. Sasnauskas K. Slibinskas R. (2011). Overexpression of human virus surface glycoprotein precursors induces cytosolic unfolded protein response in *Saccharomyces cerevisiae*. Microb. Cell Fact. 10:37. 10.1186/1475-2859-10-37 21595909 PMC3120639

[B11] De Schutter K. Lin Y. C. Tiels P. Van Hecke A. Glinka S. Weber-Lehmann J.. (2009). Genome sequence of the recombinant protein production host *Pichia pastoris*. Nat. Biotechnol. 27, 561–566. 10.1038/nbt.1544 19465926

[B12] Dimcheff D. E. Faasse M. A. McAtee F. J. Portis J. L. (2004). Endoplasmic reticulum (ER) stress induced by a neurovirulent mouse retrovirus is associated with prolonged BiP binding and retention of a viral protein in the ER. J. Biol. Chem. 279, 33782–33790. 10.1074/jbc.M403304200 15178688

[B13] Dobson C. M. (2004). Principles of protein folding, misfolding and aggregation. Semin. Cell Dev. Biol. 15, 3–16. 10.1016/j.semcdb.2003.12.008 15036202

[B14] Dragosits M. Stadlmann J. Albiol J. Baumann K. Maurer M. Gasser B.. (2009). The effect of temperature on the proteome of recombinant *Pichia pastoris*. J. Proteome Res. 8, 1380–1392. 10.1021/pr8007623 19216534

[B15] Farzadfard F. Perli S. D. Lu T. K. (2013). Tunable and multifunctional eukaryotic transcription factors based on CRISPR/Cas. ACS Synth. Biol. 2, 604–613. 10.1021/sb400081r 23977949 PMC3805333

[B16] Ferreira T. Mason A. B. Pypaert M. Allen K. E. Slayman C. W. (2002). Quality control in the yeast secretory pathway: a MISFOLDED PMA1 H+-ATPase REVEALS TWO CHECKPOINTS. J. Biol. Chem. 277, 21027–21040. 10.1074/jbc.M112281200 11877403

[B17] Gasser B. Saloheimo M. Rinas U. Dragosits M. Rodríguez-Carmona E. Baumann K.. (2008). Protein folding and conformational stress in microbial cells producing recombinant proteins: a host comparative overview. Microb. Cell Fact. 7:11. 10.1186/1475-2859-7-11 18394160 PMC2322954

[B18] Gasser B. Sauer M. Maurer M. Stadlmayr G. Mattanovich D. (2007). Transcriptomics-based identification of novel factors enhancing heterologous protein secretion in yeasts. Appl. Environ. Microbiol. 73, 6499–6507. 10.1128/aem.01196-07 17766460 PMC2075068

[B19] Ghaemmaghami S. Huh W. K. Bower K. Howson R. W. Belle A. Dephoure N.. (2003). Global analysis of protein expression in yeast. Nature 425, 737–741. 10.1038/nature02046 14562106

[B20] Giaever G. Chu A. M. Ni L. Connelly C. Riles L. Véronneau S.. (2002). Functional profiling of the *Saccharomyces cerevisiae* genome. Nature 418, 387–391. 10.1038/nature00935 12140549

[B21] Giaever G. Nislow C. (2014). The yeast deletion collection: a decade of functional genomics. Genetics 197, 451–465. 10.1534/genetics.114.161620 24939991 PMC4063906

[B22] Gleeson M. A. White C. E. Meininger D. P. Komives E. A. (1998). Generation of protease-deficient strains and their use in heterologous protein expression. Methods Mol. Biol. 103, 81–94. 10.1385/0-89603-421-6:81 9680635

[B23] Graf A. Gasser B. Dragosits M. Sauer M. Leparc G. G. Tüchler T.. (2008). Novel insights into the unfolded protein response using *Pichia pastoris* specific DNA microarrays. BMC Genomics 9:390. 10.1186/1471-2164-9-390 18713468 PMC2533675

[B24] Harmsen M. Bruyne M. Raué H. Maat J. (1996). Overexpression of binding protein and disruption of the PMR1 gene synergistically stimulate secretion of bovine prochymosin but not plant thaumatin in yeast. Appl. Microbiol. Biotechnol. 46, 365–370. 10.1007/BF00166231 8987725

[B25] Haslbeck M. Braun N. Stromer T. Richter B. Model N. Weinkauf S.. (2004). Hsp42 is the general small heat shock protein in the cytosol of *Saccharomyces cerevisiae*. EMBO J. 23, 638–649. 10.1038/sj.emboj.7600080 14749732 PMC1271810

[B26] Heinisch J. J. Lorberg A. Schmitz H. P. Jacoby J. J. (1999). The protein kinase C-mediated MAP kinase pathway involved in the maintenance of cellular integrity in *Saccharomyces cerevisiae*. Mol. Microbiol. 32, 671–680. 10.1046/j.1365-2958.1999.01375.x 10361272

[B27] Hollinger F. B. Liang T. J. (2001). Hepatitis B Virus. Fields Virology, 4th Edn. Philadelphia, PA: Lippincott-Raven Publishers.

[B28] Hou J. Tyo K. E. Liu Z. Petranovic D. Nielsen J. (2012a). Metabolic engineering of recombinant protein secretion by *Saccharomyces cerevisiae*. FEMS Yeast Res. 12, 491–510. 10.1111/j.1567-1364.2012.00810.x 22533807

[B29] Hou J. Tyo K. Liu Z. Petranovic D. Nielsen J. (2012b). Engineering of vesicle trafficking improves heterologous protein secretion in *Saccharomyces cerevisiae*. Metab. Eng. 14, 120–127. 10.1016/j.ymben.2012.01.002 22265825

[B30] Idiris A. Tohda H. Kumagai H. Takegawa K. (2010). Engineering of protein secretion in yeast: strategies and impact on protein production. Appl. Microbiol. Biotechnol. 86, 403–417. 10.1007/s00253-010-2447-0 20140428

[B31] Iwata Y. Koizumi N. (2012). Plant transducers of the endoplasmic reticulum unfolded protein response. Trends Plant Sci. 17, 720–727. 10.1016/j.tplants.2012.06.014 22796463

[B32] Jakočiūnas T. Bonde I. Herrgård M. Harrison S. J. Kristensen M. Pedersen L. E.. (2015). Multiplex metabolic pathway engineering using CRISPR/Cas9 in *Saccharomyces cerevisiae*. Metab. Eng. 28, 213–222. 10.1016/j.ymben.2015.01.008 25638686

[B33] Jones E. W. (1991). Tackling the protease problem in *Saccharomyces cerevisiae*. Methods Enzymol. 194, 428–453. 10.1016/0076-6879(91)94034-A 2005802

[B34] Kim B. Du J. Eriksen D. T. Zhao H. (2013). Combinatorial design of a highly efficient xylose-utilizing pathway in *Saccharomyces cerevisiae* for the production of cellulosic biofuels. Appl. Environ. Microbiol. 79, 931–941. 10.1128/AEM.02736-12 23183982 PMC3568569

[B35] Klausner R. D. (1989). Architectural editing: determining the fate of newly synthesized membrane proteins. New Biol. 1, 3–8. 2488271

[B36] Krogan N. J. Cagney G. Yu H. Zhong G. Guo X. Ignatchenko A.. (2006). Global landscape of protein complexes in the yeast *Saccharomyces cerevisiae*. Nature 440, 637–643. 10.1038/nature04670 16554755

[B37] Larson M. H. Gilbert L. A. Wang X. Lim W. A. Weissman J. S. Qi L. S. (2013). CRISPR interference (CRISPRi) for sequence-specific control of gene expression. Nat. Protoc. 8, 2180–2196. 10.1038/nprot.2013.132 24136345 PMC3922765

[B38] Lazar C. Uta M. Branza-Nichita N. (2014). Modulation of the unfolded protein response by the human hepatitis B virus. Front. Microbiol. 5:433. 10.3389/fmicb.2014.00433 25191311 PMC4137222

[B39] Lee K. Tirasophon W. Shen X. Michalak M. Prywes R. Okada T. (2002). IRE1-mediated unconventional mRNA splicing and S2P-mediated ATF6 cleavage merge to regulate XBP1 in signaling the unfolded protein response. Genes Dev. 16, 452–466. 10.1101/gad.964702 11850408 PMC155339

[B40] Liu L. Feizi A. Osterlund T. Hjort C. Nielsen J. (2014). Genome-scale analysis of the high-efficient protein secretion system of *Aspergillus oryzae*. BMC Syst. Biol. 8:73 10.1186/1752-0509-8-73 24961398 PMC4086290

[B41] Love K. R. Politano T. J. Panagiotou V. Jiang B. Stadheim T. A. Love J. C.. (2012). Systematic single-cell analysis of *Pichia pastoris* reveals secretory capacity limits productivity. PLoS ONE 7:e37915. 10.1371/journal.pone.0037915 22685548 PMC3369916

[B42] Ma Y. Hendershot L. M. (2001). The unfolding tale of the unfolded protein response. Cell 107, 827–830 10.1016/S0092-8674(01)00623-7 11779459

[B43] Mattanovich D. Gasser B. Hohenblum H. Sauer M. (2004). Stress in recombinant protein producing yeasts. J. Biotechnol. 113, 121–135. 10.1016/j.jbiotec.2004.04.035 15380652

[B44] Mattanovich D. Graf A. Stadlmann J. Dragosits M. Redl A. Maurer M.. (2009). Genome, secretome and glucose transport highlight unique features of the protein production host *Pichia pastoris*. Microb. Cell Fact. 8:29. 10.1186/1475-2859-8-29 19490607 PMC2702363

[B45] Mattia A. Merker R. (2008). Regulation of probiotic substances as ingredients in foods: premarket approval or “generally recognized as safe” notification. Clin. Infect. Dis. 46 (Suppl. 2), S115–S118; discussion: S144–S151. 10.1086/523329 18181714

[B46] Mulder H. J. Saloheimo M. Penttila M. Madrid S. M. (2004). The transcription factor HACA mediates the unfolded protein response in *Aspergillus niger* and up-regulates its own transcription. Mol. Genet. Genomics 271, 130–140. 10.1007/s00438-003-0965-5 14730445

[B47] Nielsen J. (2013). Production of biopharmaceutical proteins by yeast: advances through metabolic engineering. Bioengineered 4, 207–211. 10.4161/bioe.22856 23147168 PMC3728191

[B48] Palomares L. A. Estrada-Mondaca S. Ramirez O. T. (2004). Production of recombinant proteins: challenges and solutions. Methods Mol. Biol. 267, 15–52. 10.1385/1-59259-774-2:015 15269414

[B49] Park S. K. Xiao H. Lei M. (2014). Nuclear FKBPs, Fpr3 and Fpr4 affect genome-wide genes transcription. Mol. Genet. Genomics 289, 125–136. 10.1007/s00438-013-0794-0 24297734

[B50] Payne T. Finnis C. Evans L. R. Mead D. J. Avery S. V. Archer D. B.. (2008). Modulation of chaperone gene expression in mutagenized *Saccharomyces cerevisiae* strains developed for recombinant human albumin production results in increased production of multiple heterologous proteins. Appl. Environ. Microbiol. 74, 7759–7766. 10.1128/aem.01178-08 18931293 PMC2607181

[B51] Qi L. S. Larson M. H. Gilbert L. A. Doudna J. A. Weissman J. S. Arkin A. P.. (2013). Repurposing CRISPR as an RNA-guided platform for sequence-specific control of gene expression. Cell 152, 1173–1183. 10.1016/j.cell.2013.02.022 23452860 PMC3664290

[B52] Resina D. Bollók M. Khatri N. K. Valero F. Neubauer P. Ferrer P. (2007). Transcriptional response of *P. pastoris* in fed-batch cultivations to Rhizopus oryzae lipase production reveals UPR induction. Microb. Cell Fact. 6:21. 10.1186/1475-2859-6-21 17634115 PMC1950523

[B53] Robinson A. Hines V. Wittrup K. (1994). Protein disulfide isomerase overexpression increases secretion of foreign proteins in *Saccharomyces cerevisiae*. Biotechnology 12, 381–384. 10.1038/nbt0494-381 7764684

[B54] Robson G. D. (2007). Exploitation of Fungi. Cambridge, UK: Cambridge University Press.

[B55] Ron D. Walter P. (2007). Signal integration in the endoplasmic reticulum unfolded protein response. Nat. Rev. Mol. Cell Biol. 8, 519–529. 10.1038/nrm2199 17565364

[B56] Rosochacki S. J. Matejczyk M. (2002). Green fluorescent protein as a molecular marker in microbiology. Acta Microbiol. Pol. 51, 205–216. 12588095

[B57] Schekman R. (2010). Charting the secretory pathway in a simple eukaryote. Mol. Biol. Cell 21, 3781–3784. 10.1091/mbc.e10-05-0416 21079008 PMC2982102

[B58] Schroder M. Kaufman R. J. (2005). ER stress and the unfolded protein response. Mutat. Res. 569, 29–63. 10.1016/j.mrfmmm.2004.06.056 15603751

[B59] Shetty R. Lizarazo M. Rettberg R. Knight T. F. (2011). Assembly of BioBrick standard biological parts using three antibiotic assembly. Methods Enzymol. 498, 311–326. 10.1016/B978-0-12-385120-8.00013-9 21601683

[B60] Shusta E. Raines R. Plückthun A. Wittrup K. (1998). Increasing the secretory capacity of *Saccharomyces cerevisiae* for production of single-chain antibody fragments. Nat. Biotechnol. 16, 773–777. 10.1038/nbt0898-773 9702778

[B61] Soromani C. Zeng N. Hollemeyer K. Heinzle E. Klein M. C. Tretter T.. (2012). N-acetylation and phosphorylation of Sec complex subunits in the ER membrane. BMC Cell Biol. 13:34. 10.1186/1471-2121-13-34 23237413 PMC3541991

[B62] Stadlmayr G. Benakovitsch K. Gasser B. Mattanovich D. Sauer M. (2010). Genome-scale analysis of library sorting (GALibSo): isolation of secretion enhancing factors for recombinant protein production in *Pichia pastoris*. Biotechnol. Bioeng. 105, 543–555. 10.1002/bit.22573 19816964

[B63] Stevens F. J. Argon Y. (1999). Protein folding in the ER. Semin. Cell Dev. Biol. 10, 443–454. 10.1006/scdb.1999.0315 10597627

[B64] Travers K. J. Patil C. K. Wodicka L. Lockhart D. J. Weissman J. S. Walter P. (2000). Functional and genomic analyses reveal an essential coordination between the unfolded protein response and ER-associated degradation. Cell 101, 249–258. 10.1016/S0092-8674(00)80835-1 10847680

[B65] Urade R. (2007). Cellular response to unfolded proteins in the endoplasmic reticulum of plants. FEBS J. 274, 1152–1171. 10.1111/j.1742-4658.2007.05664.x 17257164

[B66] Wagner C. Dietz M. Wittmann J. Albrecht A. Schuller H. J. (2001). The negative regulator Opi1 of phospholipid biosynthesis in yeast contacts the pleiotropic repressor Sin3 and the transcriptional activator Ino2. Mol. Microbiol. 41, 155–166. 10.1046/j.1365-2958.2001.02495.x 11454208

[B67] Wang Q. Chang A. (1999). Eps1, a novel PDI-related protein involved in ER quality control in yeast. EMBO J. 18, 5972–5982. 10.1093/emboj/18.21.5972 10545109 PMC1171663

[B68] Wildt S. Gerngross T. U. (2005). The humanization of N-glycosylation pathways in yeast. Nat. Rev. Microbiol. 3, 119–128. 10.1038/nrmicro1087 15685223

[B69] Winzeler E. A. Shoemaker D. D. Astromoff A. Liang H. Anderson K. Andre B.. (1999). Functional characterization of the *S. cerevisiae* genome by gene deletion and parallel analysis. Science 285, 901–906. 10.1126/science.285.5429.901 10436161

[B70] Ye C. Dickman M. B. Whitham S. A. Payton M. Verchot J. (2011). The unfolded protein response is triggered by a plant viral movement protein. Plant Physiol. 156, 741–755. 10.1104/pp.111.174110 21474436 PMC3177272

[B71] Young B. P. Craven R. A. Reid P. J. Willer M. Stirling C. J. (2001). Sec63p and Kar2p are required for the translocation of SRP-dependent precursors into the yeast endoplasmic reticulum *in vivo*. EMBO J. 20, 262–271. 10.1093/emboj/20.1.262 11226176 PMC140194

[B72] Zhang B. Chang A. Kjeldsen T. B. Arvan P. (2001). Intracellular retention of newly synthesized insulin in yeast is caused by endoproteolytic processing in the Golgi complex. J. Cell Biol. 153, 1187–1198. 10.1083/jcb.153.6.1187 11402063 PMC2192022

[B73] Zhang K. Kaufman R. J. (2006). The unfolded protein response: a stress signaling pathway critical for health and disease. Neurology 66, S102–S109. 10.1212/01.wnl.0000192306.98198.ec 16432136

[B74] Zhang L. Wang A. (2012). Virus-induced ER stress and the unfolded protein response. Front. Plant Sci. 3:293. 10.3389/fpls.2012.00293 23293645 PMC3531707

