# Supplementary material for: Systematic Optimization of Protein Secretory Pathways in Saccharomyces cerevisiae to Increase Expression of Hepatitis B Small Antigen
Source: Front Microbiol. 2017 May 16;8:875. doi: 10.3389/fmicb.2017.00875 (PMC5432677; doi:10.3389/fmicb.2017.00875)
Supplement: Table S1 — Sequences of the constructs and primers used in this study. [file Table1.DOCX]

**Table 1S** Sequences of the constructs and primers used in this study.

| **Name** | **Sequence (5’-3’)** |
| --- | --- |
| HBsAg-eGFP | ATGGAGAACATCACATCAGGATTCCTAGGACCCCTGCTCGTGTTACAGGCGGGGTTTTTCTTGTTGACAAGAATCCTCACAATACCGCAGAGTCTAGACTCGTGGTGGACTTCTCTCAATTTTCTAGGGGGATCACCCGTGTGTCTTGGCCAAAATTCGCAGTCCCCAACCTCCAATCACTCACCAACCTCCTGTCCTCCAATTTGTCCTGGTTATCGCTGGATGTGTCTGAGGCGTTTTATCATATTCCTCTTCATCCTGCTGCTATGCCTCATCTTCTTATTGGTTCTTCTGGATTATCAAGGTATGTTGCCCGTTTGTCCTCTAATTCCAGGATCAACAACAACCAATACGGGACCATGCAAAACCTGCACGACTCCTGCTCAAGGCAACTCTATGTTTCCCTCATGTTGCTGTACAAAACCTACGGATGGAAATTGCACCTGTATTCCCATCCCATCGTCCTGGGCTTTCGCAAAATACCTATGGGAGTGGGCCTCAGTCCGTTTCTCTTGGCTCAGTTTACTAGTGCCATTTGTTCAGTGGTTCGTAGGGCTTTCCCCCACTGTTTGGCTTTCAGCTATATGGATGATGTGGTATTGGGGGCCAAGTCTGTACAGCATCGTGAGTCCCTTTATACCGCTGTTACCAATTTTCTTTTGTCTCTGGGTATACATT**GGTGAAAATTTGTATTTTCAATCTGGTGGT**ATGTCTAAAGGTGAAGAATTATTCACTGGTGTTGTCCCAATTTTGGTTGAATTAGATGGTGATGTTAATGGTCACAAATTTTCTGTCTCCGGTGAAGGTGAAGGTGATGCTACTTACGGTAAATTGACCTTAAAATTTATTTGTACTACTGGTAAATTGCCAGTTCCATGGCCAACCTTAGTCACTACTTTAACTTATGGTGTTCAATGTTTTTCTAGATACCCAGATCATATGAAACAACATGACTTTTTCAAGTCTGCCATGCCAGAAGGTTATGTTCAAGAAAGAACTATTTTTTTCAAAGATGACGGTAACTACAAGACCAGAGCTGAAGTCAAGTTTGAAGGTGATACCTTAGTTAATAGAATCGAATTAAAAGGTATTGATTTTAAAGAAGATGGTAACATTTTAGGTCACAAATTGGAATACAACTATAACTCTCACAATGTTTACATCATGGCTGACAAACAAAAGAATGGTATCAAAGTTAACTTCAAAATTAGACACAACATTGAAGATGGTTCTGTTCAATTAGCTGACCATTATCAACAAAATACTCCAATTGGTGATGGTCCAGTCTTGTTACCAGACAACCATTACTTATCCACTCAATCTGCCTTATCCAAAGATCCAAACGAAAAGAGAGACCACATGGTCTTGTTAGAATTTGTTACTGCTGCTGGTATTACCCATGGTATGGATGAATTGTACAAATAA  The underlined sequence stands for the flexible protein linker between HBsAg and eGFP |
| gRNA  expression  cassette  (pRPR1_ _HindIII_SDS  _gRNA backbone_ _RPR1t | GAGCTCGGGGGATCTGCCAATTGAACATAACATGGTAGTTACATATACTAGTAATATGGTTCGGCACACATTAAAAGTATAAAAACTATCTGAATTACGAATTACATATATTGGTCATAAAAATCAATCAATCATCGTGTGTTTTATATGTCTCTTATCTAAGTATAAGAATATCCATAGTTAATATTCACTTACGCTACCTTTTAACCTGTAATCATTGTCAACAGGATATGTTAACGACCCACATTGATAAACGCTAGTATTTCTTTTTCCTCTTCTTATTGGCCGGCTGTCTCTATACTCCCCTATAGTCTGTTTCTTTTCGTTTCGATTGTTTTACGTTTGAGGCCTCGTGGCGCACATGGTACGCTGTGGTGCTCGCGGCTGGGAACGAAACTCTGGGAGCTGCGATTGGCAGAAGCTT**NNNNNNNNNNNNNNNNNNNN**GTTTTAGAGCTAGAAATAGCAAGTTAAAATAAGGCTAGTCCGTTATCAACTTGAAAAAGTGGCACCGAGTCGGTGCTTTTTTCTCGAGCCATATCCAACTTCCAATTTAATCTTTCTTTTTTAATTTTCACTTATTTGCGATACAGAAAGAAAAAAGCGATAGTAACTATTGAATTTTGTTTGGATTTGGTTAGATTAGATATGGTTTCTCTTTATATTTACATGCTAAAAATGGGCTACACCAGAGATACATAATTAGATATATATACGCCAGTACACCTTATCGGCCCAAGCCTTGTCCCAAGGCAGCGTTTTGTTCTTGGAAACGCTGCCCTACACGTTCGCTATGCTTCAAGAACTTTTCTGAGCACTTCATGATGCATGTTTGTTCCTTATTGGTTAGCTTTGATGTTGTGAAGTCATTGACACAGTCTGTGAAACATCTTTCTACCAGATTAGAGTACAAACGCATGAAATCCTTCATTTGCTTTTGTTCCACTACTTTTTGGAACTCTTGTTGTTCTTTGGTACC  Where **NNNNNNNNNNNNNNNNNNNN** is one of the following: |
| gRNA_Y1 | AGAATGAGCAACGAAGATTA |
| gRNA_Y2 | TTATTTAAGATAGTTCTTAT |
| gRNA_Y3 | ATAGTTCTTATCGGCGACTC |
| gRNA_S1 | ATGTCAAGCCCAACTCCTCC |
| gRNA_S2 | TCAAGCCCAACTCCTCCAGG |
| gRNA_S3 | TACTTTGCAAAAGAGAAAAC |
| gRNA_H1 | AACGCATTATCCAACCAAAC |
| gRNA_H2 | CCAACCAAACTGGCCAGAGA |
| gRNA_H3 | ACTGGCCAGAGAGGGCAGCA |
| **Primers** | **Sequence (5’-3’)** |
| TEF1p_F | GACGGTATCGATAAGCTTGATATCGAATTCCTGCAGCCCATAGCTTCAAAATGTTTCTAC |
| TEF1p_R_HBsAg-GFP | GAGCAGGGGTCCTAGGAATCCTGATGTGATGTTCTCCATTTTGTAATTAAAACTTAGATT |
| HBsAg-GFP_F | AAAGAAAGCATAGCAATCTAATCTAAGTTTTAATTACAAAATGGAGAACATCACATCAGG |
| HBsAg-GFP_R | ATATAAAAGATATGCAACTAGAAAAGTCTTATCAATCTCCTTATTTGTACAATTCATCCA |
| TEF1t_F_HBsAg-GFP | TGCTGGTATTACCCATGGTATGGATGAATTGTACAAATAAGGAGATTGATAAGACTTTTC |
| TEF1t_R | CCACCGCGGTGGCGGCCGCTCTAGAACTAGTGGATCCCCCGATAGCGCCGATCAAAGTAT |
| TEF1p_R_IRE1 | GCAGTGTCAATACTAACATGTTTCTTCGAAGTAGACGCATTTTGTAATTAAAACTTAGAT |
| IRE1_F | AAAGAAAGCATAGCAATCTAATCTAAGTTTTAATTACAAAATGCGTCTACTTCGAAGAAA |
| IRE1_R | ATATAAAAGATATGCAACTAGAAAAGTCTTATCAATCTCCTTATGAATACAAAAATTCAC |
| TEF1t_F_IRE1 | AAGTGACGATCAAATTTTACGTGAATTTTTGTATTCATAAGGAGATTGATAAGACTTTTC |
| TEF1p_F_BCK1 | GTGTATGTGCTGTCCCCGCTATTTTCCTCAAAAAGGGCATTTTGTAATTAAAACTTAGAT |
| BCK1_F | AAAGAAAGCATAGCAATCTAATCTAAGTTTTAATTACAAAATGCCCTTTTTGAGGAAAAT |
| BCK1_R | ATATAAAAGATATGCAACTAGAAAAGTCTTATCAATCTCCTTATTCAGTTTTATTCTCCT |
| TEF1t_F_ BCK1 | AAAATTAAGGATAACCTCTCAGGAGAATAAAACTGAATAAGGAGATTGATAAGACTTTTC |
| TEF1p_F_OPI1 | CCTCTTCCTCTGATAATCCTAAACGTTGATTTTCAGACATTTTGTAATTAAAACTTAGAT |
| OPI1_F | AAAGAAAGCATAGCAATCTAATCTAAGTTTTAATTACAAAATGTCTGAAAATCAACGTTT |
| OPI1_R | ACGTGGATAGCAAGGACTAAGGAGATTGATAAGACTTTTCTAGTTGCATATCTTTTATAT |
| TEF1t_F_ OPI1 | CGTAAAGCCCTCTCAGGACAACGTGGATAGCAAGGACTAAGGAGATTGATAAGACTTTTC |
| TEF1p_F_SSA4 | AATAGGTTGTACCTAAATCAATACCAACAGCTTTTGACATTTTGTAATTAAAACTTAGAT |
| SSA4_F | AAAGAAAGCATAGCAATCTAATCTAAGTTTTAATTACAAAATGTCAAAAGCTGTTGGTAT |
| SSA4_R | ATATAAAAGATATGCAACTAGAAAAGTCTTATCAATCTCCTTAATCAACCTCTTCAACCG |
| TEF1t_F_ SSA4 | AGCACCAGACAACGGCCCAACGGTTGAAGAGGTTGATTAAGGAGATTGATAAGACTTTTC |
| TEF1p_F_EPS1 | AGAAGAAGGTAACTACGAGCCTTTTCAGATTCATTTTCATTTTGTAATTAAAACTTAGAT |
| EPS1_F | AAAGAAAGCATAGCAATCTAATCTAAGTTTTAATTACAAAATGAAAATGAATCTGAAAAG |
| EPS 1_R | ATATAAAAGATATGCAACTAGAAAAGTCTTATCAATCTCCTTAATCTTGATTTTTTTTTT |
| TEF1t_F_EPS1 | TATCCTAGGAAATATGGAGAAAAAAAAAAATCAAGATTAAGGAGATTGATAAGACTTTTC |
