# Supplementary material for: Systematic Optimization of Protein Secretory Pathways in Saccharomyces cerevisiae to Increase Expression of Hepatitis B Small Antigen
Source: Front Microbiol. 2017 May 16;8:875. doi: 10.3389/fmicb.2017.00875 (PMC5432677; doi:10.3389/fmicb.2017.00875)
Supplement: Table S2 — The plasmids constructed in this study. [file Table2.DOCX]

**Table 2S.** The plasmids constructed in this study.

| **Name** | **Description** | **Reference** |
| --- | --- | --- |
| pRPR1_gRNA_handle_RPR1t | pRS425- RPR1p_gRNA_handle_RPR1t | Farzadfard et al. (2013)^1^ |
| pdCas9 | pRS413- TEF1p-dCas9-TEF1t | This study |
| pHBV-GFP | pRS416- TEF1p-HBsAg-eGFP-TEF1t | This study |
| pOPI1 | pRS415- ENO2p- OPI1-ENO2t | This study |
| pEPS1 | pRS415- ENO2p- EPS1-ENO2t | This study |
| pSSA4 | pRS415- ENO2p- SSA4-ENO2t | This study |
| pBCK1 | pRS415- ENO2p- BCK1-ENO2t | This study |
| pIRE1 | pRS415- ENO2p- IRE1-ENO2t | This study |
| pIRE1- HBV-GFP | pRS416- ENO2p- IRE1-ENO2t- TEF1p-HBsAg-eGFP-TEF1t | This study |
| pgRNA_Y1 | pRS425- RPR1p_gRNAY1_RPR1t | This study |
| pgRNA_Y2 | pRS425- RPR1p_gRNAY2_RPR1t | This study |
| pgRNA_Y3 | pRS425- RPR1p_gRNAY3_RPR1t | This study |
| pgRNA_S1 | pRS425- RPR1p_gRNAS1_RPR1t | This study |
| pgRNA_S2 | pRS425- RPR1p_gRNAS2_RPR1t | This study |
| pgRNA_S3 | pRS425- RPR1p_gRNAS3_RPR1t | This study |
| pgRNA_H1 | pRS425- RPR1p_gRNAH1_RPR1t | This study |
| pgRNA_H2 | pRS425- RPR1p_gRNAH2_RPR1t | This study |
| pgRNA_H3 | pRS425- RPR1p_gRNAH3_RPR1t | This study |
| pgRNAY1S1H1 | pRS415-RPR1p_gRNAY1_RPR1t_RPR1p_gRNAS1_RPR1t_RPR1p_gRNAH1_RPR1t | This study |
| pgRNAY1S1H2 | pRS415-RPR1p_gRNAY1_RPR1t_RPR1p_gRNAS1_RPR1t_RPR1p_gRNAH2_RPR1t | This study |
| pgRNAY1S1H3 | pRS415-RPR1p_gRNAY1_RPR1t_RPR1p_gRNAS1_RPR1t_RPR1p_gRNAH3_RPR1t | This study |
| pgRNAY1S2H1 | pRS415-RPR1p_gRNAY1_RPR1t_RPR1p_gRNAS2_RPR1t_RPR1p_gRNAH1_RPR1t | This study |
| pgRNAY1S2H2 | pRS415-RPR1p_gRNAY1_RPR1t_RPR1p_gRNAS2_RPR1t_RPR1p_gRNAH2_RPR1t | This study |
| pgRNAY1S2H3 | pRS415-RPR1p_gRNAY1_RPR1t_RPR1p_gRNAS2_RPR1t_RPR1p_gRNAH3_RPR1t | This study |
| pgRNAY1S3H1 | pRS415-RPR1p_gRNAY1_RPR1t_RPR1p_gRNAS3_RPR1t_RPR1p_gRNAH1_RPR1t | This study |
| pgRNAY1S3H2 | pRS415-RPR1p_gRNAY1_RPR1t_RPR1p_gRNAS3_RPR1t_RPR1p_gRNAH2_RPR1t | This study |
| pgRNAY1S3H3 | pRS415-RPR1p_gRNAY1_RPR1t_RPR1p_gRNAS3_RPR1t_RPR1p_gRNAH3_RPR1t | This study |
| pgRNAY2S1H1 | pRS415-RPR1p_gRNAY2_RPR1t_RPR1p_gRNAS1_RPR1t_RPR1p_gRNAH1_RPR1t | This study |
| pgRNAY2S1H2 | pRS415-RPR1p_gRNAY2_RPR1t_RPR1p_gRNAS1_RPR1t_RPR1p_gRNAH2_RPR1t | This study |
| pgRNAY2S1H3 | pRS415-RPR1p_gRNAY2_RPR1t_RPR1p_gRNAS1_RPR1t_RPR1p_gRNAH3_RPR1t | This study |
| pgRNAY2S2H1 | pRS415-RPR1p_gRNAY2_RPR1t_RPR1p_gRNAS2_RPR1t_RPR1p_gRNAH1_RPR1t | This study |
| pgRNAY2S2H2 | pRS415-RPR1p_gRNAY21_RPR1t_RPR1p_gRNAS2_RPR1t_RPR1p_gRNAH2_RPR1t | This study |
| pgRNAY2S2H3 | pRS415-RPR1p_gRNAY2_RPR1t_RPR1p_gRNAS2_RPR1t_RPR1p_gRNAH3_RPR1t | This study |
| pgRNAY2S3H1 | pRS415-RPR1p_gRNAY2_RPR1t_RPR1p_gRNAS3_RPR1t_RPR1p_gRNAH1_RPR1t | This study |
| pgRNAY2S3H2 | pRS415-RPR1p_gRNAY2_RPR1t_RPR1p_gRNAS3_RPR1t_RPR1p_gRNAH2_RPR1t | This study |
| pgRNAY2S3H3 | pRS415-RPR1p_gRNAY2_RPR1t_RPR1p_gRNAS3_RPR1t_RPR1p_gRNAH3_RPR1t | This study |
| pgRNAY3S1H1 | pRS415-RPR1p_gRNAY3_RPR1t_RPR1p_gRNAS1_RPR1t_RPR1p_gRNAH1_RPR1t | This study |
| pgRNAY3S1H2 | pRS415-RPR1p_gRNAY3_RPR1t_RPR1p_gRNAS1_RPR1t_RPR1p_gRNAH2_RPR1t | This study |
| pgRNAY3S1H3 | pRS415-RPR1p_gRNAY3_RPR1t_RPR1p_gRNAS1_RPR1t_RPR1p_gRNAH3_RPR1t | This study |
| pgRNAY3S2H1 | pRS415-RPR1p_gRNAY3_RPR1t_RPR1p_gRNAS2_RPR1t_RPR1p_gRNAH1_RPR1t | This study |
| pgRNAY3S2H2 | pRS415-RPR1p_gRNAY3_RPR1t_RPR1p_gRNAS2_RPR1t_RPR1p_gRNAH2_RPR1t | This study |
| pgRNAY3S2H3 | pRS415-RPR1p_gRNAY3_RPR1t_RPR1p_gRNAS2_RPR1t_RPR1p_gRNAH3_RPR1t | This study |
| pgRNAY3S3H1 | pRS415-RPR1p_gRNAY3_RPR1t_RPR1p_gRNAS3_RPR1t_RPR1p_gRNAH1_RPR1t | This study |
| pgRNAY3S3H2 | pRS415-RPR1p_gRNAY3_RPR1t_RPR1p_gRNAS3_RPR1t_RPR1p_gRNAH2_RPR1t | This study |
| pgRNAY3S3H3 | pRS415-RPR1p_gRNAY3_RPR1t_RPR1p_gRNAS3_RPR1t_RPR1p_gRNAH3_RPR1t | This study |
