# Supplementary material for: Systematic Optimization of Protein Secretory Pathways in Saccharomyces cerevisiae to Increase Expression of Hepatitis B Small Antigen
Source: Front Microbiol. 2017 May 16;8:875. doi: 10.3389/fmicb.2017.00875 (PMC5432677; doi:10.3389/fmicb.2017.00875)
Supplement: Table S3 — Genes used for systematic investigation of protein secretory pathway in S. cerevisiae. [file Table3.DOCX]

**Table 3S.** Genes used for systematic investigation of protein secretory pathway in *S. cerevisiae*.

| **Classification** | **Gene ontology** | **Associated gene(s)Genes** |
| --- | --- | --- |
| ER-associated degradation | ER-associated ubiquitin-dependent protein catabolic process | ADD37, ATG19, BST1, CDC48, CNE1, CUE1, DER1, DFM1, DSK2, EPS1, HLJ1, HRD1,HRD3, HUL5, JEM1, KAR2,LCL2, MNL1, MNL2, MNS1,NPL4, PBN1, POF1, RAD23,RAD6, RPT4, SCJ1, SEC13,SEC61, SHP1, SSM4, UBC1,UBC6, UBC7, UBR1, UBX2,UBX4, UFD1, UFD2, USA1,VMS1, YDJ1, YDR161W, YOS9 |
| Protein folding | *'de novo'*protein folding | HSC82, HSP60, HSP82, MDJ1, ROT1, YDJ1 |
|  | protein folding in endoplasmic reticulum | EMC1, EMC2, EMC3, EMC4, EMC5, EMC6, EMP65, ERO1, HSP104, JEM1, OSM1, SCJ1, SLP1 |
|  | ATP-independent chaperone mediated protein folding | GET3 |
|  | 'de novo' cotranslational protein folding | BTT1, EGD1, EGD2, SSB1, SSB2 |
|  | protein folding | AHA1, ALF1, APJ1, BTN2, CAJ1, CCT2, CCT3, CCT4, CCT5, CCT6, CCT7, CCT8, CHS7, CIN1, CIN2, CIN4, CNE1, CNS1, CPR1, CPR2, CPR3, CPR4 , CPR5 , CPR6 , CPR7 ,CPR8 , CUR1, CWC27, ECM10, EPS1, ERJ5 , EUG1 , FLC1 ,FLC2, FMO1, FPR1, FPR2, FPR3, FPR4, GIM3, GIM4, GIM5, GSF2, HCH1, HSC82, HSP10, HSP26, HSP60, HSP82, JAC1 , MCX1 , MDJ1 , MGE1 ,MIA40, MPD1, MPD2, PAC10, PAC2 , PDI1 , PFD1, PHB1, PHB2, PHO86, PIH1, PLP1, PLP2, RDN25-1, RDN252, ROT1, SBA1, SCJ1, SHR3, SIS1, SNL1, SSA1, SSA2, SSA3, SSA4, SSC1, SSE1, SSE2, STI1, TAH1, TCP1, TSA1, XDJ1, YDJ1, YKE2, YME1, ZIM17, ZUO1 |
|  | protein folding in endoplasmic reticulum | EMC1, EMC2, EMC3, EMC4, EMC5 , EMC6 , EMP65 , ERO1 ,HSP104 , JEM1 , OSM1 , SCJ1 ,SLP1 |
| Translocation | SRP-dependent cotranslational protein targeting to membrane | SBH2, SEC63, SEC65, SRP101, SRP102, SRP14, SRP54, SRP68, SRP72, SSH1 |
|  | SRP-dependent cotranslational protein targeting to membrane, translocation | KAR2, SBH1, SEC61, SIL1, SSA1, SSA2, SSA3, SSA4, SSS1 |
|  | posttranslational protein targeting to membrane, translocation | SEC66, SSS1, SBH1, KAR2, LHS1, SEC72, SEC61, SEC63, SEC62 |
|  | chaperone-mediated protein folding | TSA1 |
|  | regulation of ER to Golgi vesicle-mediated transport | BRE5, UBP3 |
|  | ER to Golgi vesicle-mediated transport | AGE1, AGE2, ARF1, ATG8, BET1, BET2, BET3, BET4, BET5, BOS1, BST1, BUG1, CHS7, COG2, COG3, COP1, EMP24, EMP46, EMP47, ERD2, ERP1, ERP2, ERP4, ERP6, ERV14, ERV15, ERV25, ERV29, ERV41, ERV46, GCS1, GEA1, GEA2, GLO3, GOS1, GOT1, GRH1, GYL1, GYP5, HRR25, MRS6, NEL1, PHO86, RER1, RER2, RET2, RUD3, SEC21, SEC22, SEC23, SEC24, SEC26, SEC27, SEC28, SEC7, SED5,SFB2, SFB3, SHR3, SLY1,SLY41, SMY2, SOP4, SVP26,TCA17, TED1, TRS20, TRS23,TRS31, TRS33, TRS85, TRX1,TRX2, USO1, YIF1, YIP1, YIP3, YKT6, YOS1, YPT1 |
|  | positive regulation of SNARE complex assembly | SLY1, VPS45 |
|  | retrograde vesicle-mediated transport, Golgi to ER | BET1, COP1, DSL1, GCS1, GEA1, GEA2, GET1, GET2, GET3, GLO3, NEO1, RER1, RET2, RET3, SEC20, SEC21, SEC22, SEC27, SEC28, SEC39, SLY1, TIP20, TRX1, TRX2, UFE1, USE1, VPS74, YPT1 |
|  | regulation of retrograde vesicle-mediated transport, Golgi to ER | BRE5 |
|  | vesicle fusion with Golgi apparatus | BOS1, SEC17, SEC18, SEC22, SED5, SLY1, USO1, YIP1 |
|  | exocytosis | ACT1, EXO70, EXO84, GYL1, GYP5, HES1, KES1, KIN1, KIN2\| MSB3, MSB4, MYO3, MYO5,OSH2, OSH3, OSH6, OSH7,PMR1, RCY1, SEC1, SEC10,SEC15, SEC2, SEC3, SEC4,SEC5, SEC6, SEC8, SEC9,SEM1, SLA2, SMY1, SNC1,SNC2, SPO14, SRO7, SRO77,SWH1, SYT1, TPM1, TPM2,YPT31, YPT32 |
|  | regulation of exocytosis | GYL1, SFH5, SWF1 |
|  | positive regulation of exocytosis | CDC42, RHO3 |
|  | vesicle docking involved inexocytosis | MSO1, PEP3, PEP7, SEC1, SEC15, SEC8, SLY1, VPS33, VPS45 |
|  | vesicle tethering involved inexocytosis | EXO70, EXO84, SEC10, SEC15\| SEC3, SEC5, SEC6, SEC8 |
|  | vesicle fusion | BET1, BOS1, GOS1, NYV1, PEP7, SEC22, SEC4, SEC9, SED5, SFT1, SNC1, SNC2, SSO1, SSO2, TLG1, TLG2, VAM3, VAM7, VTI1, YKT6 |
|  | positive regulation of vesicle fusion | SEC1 |
|  | vesicle fusion with endoplasmic reticulum | SEC20, SEC22, UFE1, USE1 |
|  | vesicle fusion with Golgi apparatus | BOS1, SEC17, SEC18, SEC22, SED5, SLY1, USO1, YIP1 |
|  | Golgi vesicle fusion to target membrane | VPS45 |
| Unfolded protein response (UPR) | endoplasmic reticulum unfolded protein response | BCK1, BXI1, DCR2, DFM1, EDE1, HAC1, HRD1, IRE1, OPI1, PTC2, ROT1, SLT2, ULI1, VPS74, YHI9, YOS9 |
|  | unfolded protein binding | APJ1 , ATP10 , ATP11 , BTT1 ,CCT2 , CCT3 , CCT4 , CCT5 ,CCT6 , CCT7 , CCT8 , CDC37 ,CHS7 , CNE1 , COX20 , CPR6 ,CPR7 , ECM10 , EGD1 , EGD2 ,EPS1 , EUG1 , GET3 , GIM3 ,GIM4 , GIM5 , GSF2 , HSC82 ,HSP10 , HSP104 , HSP26 ,HSP32 , HSP33 , HSP42 , HSP60, HSP82 , IRE1 , JEM1 , KAR2 ,LHS1 , MCX1 , MDJ1 , NSG1 ,NSG2 , PAM18 , PDI1 , PET100 ,PFD1 , PHO86 , PNO1 , RBL2 ,ROT1 , SCJ1 , SHQ1 , SHR3 ,SHY1 , SIS1 , SSA1 , SSA2 ,SSA3 , SSA4 , SSB1 , SSB2 ,SSC1 , SSQ1 , SSZ1 , TCM62 ,TCP1 , TIM10 , TIM9 , TSA1 ,VMA22 , VPS45 , XDJ1 , YAR1 ,YDJ1 , YKE2 , ZUO1 |
|  | positive regulation of transcription from RNA polymerase II promoter involved in unfolded protein response | HAC1 |
|  | regulation of endoplasmic reticulum unfolded protein response | PMT1, PMT2, PMT4, YPT1 |
|  | UFP-specific transcription factor mRNA processing involved in endoplasmic reticulum unfolded protein response | MID2, SLT2, SPT20, TRL1 |
