# Supplementary material for: Systematic Optimization of Protein Secretory Pathways in Saccharomyces cerevisiae to Increase Expression of Hepatitis B Small Antigen
Source: Front Microbiol. 2017 May 16;8:875. doi: 10.3389/fmicb.2017.00875 (PMC5432677; doi:10.3389/fmicb.2017.00875)
Supplement: Table S4 — HBsAg expression in 194 yeast knockout strains (same as Figure 2A). [file Table4.DOCX]

**Table 4S.** HBsAg expression in 194 yeast knockout strains (same as Figure 2A)

| **Category** | **Gene Name** | **Fold Change** | **SD** | **P-Value** |
| --- | --- | --- | --- | --- |
| **Degradation** | UBR1 | 1.148433452 | 0. 345187 | 0.036025 |
|  | MNL2 | 1.095952258 | 0.267516 | 0.025669 |
|  | SHP1 | 1.076300923 | 0.280163 | 0.027355 |
|  | RAD23 | 1.074588789 | 0.265301 | 0.025374 |
|  | SCJ1 | 0.957136664 | 0.092134 | 0.02284 |
|  | YDJ1 | 0.94481189 | 0.317419 | 0.032323 |
|  | HRD3 | 0.938256793 | 0.204375 | 0.01725 |
|  | NMA111 | 0.923503415 | 0.341069 | 0.035476 |
|  | JEM1 | 0.904841854 | 0.26873 | 0.025831 |
|  | BST1 | 0.887914666 | 0.163335 | 0.011778 |
|  | DFM1 | 0.872673915 | 0.224251 | 0.0199 |
|  | VMS1 | 0.862060008 | 0.125641 | 0.006752 |
|  | UBX4 | 0.856997312 | 0.14052 | 0.008736 |
|  | SNA3 | 0.852589456 | 0.180724 | 0.014097 |
|  | USA1 | 0.839545561 | 0.157431 | 0.010991 |
|  | MNL1 | 0.830457793 | 0.117306 | 0.005641 |
|  | HSL7 | 0.827871309 | 0.200577 | 0.016744 |
|  | HUL5 | 0.818978216 | 0.136396 | 0.008186 |
|  | ADD37 | 0.818189566 | 0.19501 | 0.016001 |
|  | HLJ1 | 0.811350596 | 0.101469 | 0.003529 |
|  | RAD6 | 0.799923123 | 0.104454 | 0.003927 |
|  | UBC7 | 0.786329658 | 0.117456 | 0.005661 |
|  | SKP2 | 0.775685779 | 0.185583 | 0.014744 |
|  | CNE1 | 0.774899487 | 0.202614 | 0.017015 |
|  | ACL4 | 0.770931807 | 0.152724 | 0.010363 |
|  | NPL4 | 0.769732166 | 0.120043 | 0.006006 |
|  | PIM1 | 0.763938339 | 0.169397 | 0.012586 |
|  | HRD1 | 0.758254173 | 0.106894 | 0.004253 |
|  | SSM4 | 0.756375906 | 0.169034 | 0.012538 |
|  | DSK2 | 0.749696355 | 0.167287 | 0.012305 |
|  | SUE1 | 0.748657116 | 0.079311 | 0.000575 |
|  | LAP2 | 0.746996482 | 0.158156 | 0.011087 |
|  | YSP3 | 0.737910267 | 0.200042 | 0.016672 |
|  | UBX2 | 0.731217204 | 0.111168 | 0.004822 |
|  | DER1 | 0.718653292 | 0.148229 | 0.009764 |
|  | ATG19 | 0.714254738 | 0.202796 | 0.017039 |
|  | YOS9 | 0.708834568 | 0.186639 | 0.014885 |
|  | HSL1 | 0.702088611 | 0.129063 | 0.007208 |
|  | UFD2 | 0.701762193 | 0.169223 | 0.012563 |
|  | POF1 | 0.653684985 | 0.131297 | 0.007506 |
|  | MNS1 | 0.629325339 | 0.167175 | 0.01229 |
|  | CUE1 | 0.596105415 | 0.182207 | 0.014294 |
|  | EPS1 | 0.57150773 | 0.208177 | 0.017757 |
|  | NTA1 | 0.555925408 | 0.183219 | 0.0014429 |
|  | ASK10 | 0.522516854 | 0.15438 | 0.0010584 |
| Protein Folding | CPR7 | 1.40112787 | 0.247443 | 0.0022992 |
|  | MDJ1 | 1.123127859 | 0.085999 | 0.001466 |
|  | CPR5 | 1.066705373 | 0.24506 | 0.0022675 |
|  | ECM10 | 1.03228443 | 0.330677 | 0.03409 |
|  | ALF1 | 0.958586985 | 0.381306 | 0.040841 |
|  | SSB2 | 0.878330813 | 0.349393 | 0.036586 |
|  | FPR2 | 0.85314639 | 0.330958 | 0.034128 |
|  | FPR3 | 0.847765407 | 0.368491 | 0.039132 |
|  | HSP104 | 0.821002519 | 0.275955 | 0.026794 |
|  | PIH1 | 0.774375496 | 0.2312 | 0.020827 |
|  | CPR4 | 0.735987291 | 0.273645 | 0.026486 |
|  | SSB1 | 0.725066503 | 0.30467 | 0.030623 |
|  | ROT2 | 0.702569393 | 0.291167 | 0.028822 |
|  | GSF2 | 0.690054587 | 0.292522 | 0.029003 |
|  | PHO86 | 0.669053553 | 0.27992 | 0.027323 |
|  | FLC2 | 0.665985269 | 0.267186 | 0.025625 |
|  | CUR1 | 0.665530827 | 0.284518 | 0.027936 |
|  | PHB2 | 0.664646613 | 0.279709 | 0.027294 |
|  | MCX1 | 0.661171179 | 0.27324 | 0.026432 |
|  | EMC2 | 0.660771123 | 0.27716 | 0.026955 |
|  | GET3 | 0.641549751 | 0.25333 | 0.023777 |
|  | MPD2 | 0.638924657 | 0.264323 | 0.025243 |
|  | PAC10 | 0.616956437 | 0.243769 | 0.022503 |
|  | EMC4 | 0.613041979 | 0.268924 | 0.025856 |
|  | CIN1 | 0.611480613 | 0.254147 | 0.023886 |
|  | HSC82 | 0.611355213 | 0.254067 | 0.023876 |
|  | BTN2 | 0.599445838 | 0.265964 | 0.025462 |
|  | HSP26 | 0.596696061 | 0.274112 | 0.026548 |
|  | EMC5 | 0.593078644 | 0.252971 | 0.023729 |
|  | EMP65 | 0.589473816 | 0.265881 | 0.025451 |
|  | CWC27 | 0.589264549 | 0.287809 | 0.028375 |
|  | FPR4 | 0.588378732 | 0.251547 | 0.02354 |
|  | GIM3 | 0.585300392 | 0.256406 | 0.024188 |
|  | EMC3 | 0.577367716 | 0.21251 | 0.018335 |
|  | CPR2 | 0.575405307 | 0.238111 | 0.021748 |
|  | GIM5 | 0.568011362 | 0.298297 | 0.029773 |
|  | CIN2 | 0.566402063 | 0.260237 | 0.024698 |
|  | MPD1 | 0.5645105 | 0.27806 | 0.027075 |
|  | HCH1 | 0.558853982 | 0.263246 | 0.025099 |
|  | AHA1 | 0.55690186 | 0.216333 | 0.018844 |
|  | EGD2 | 0.554350616 | 0.283283 | 0.027771 |
|  | CPR1 | 0.552100732 | 0.245825 | 0.022777 |
|  | EUG1 | 0.542252785 | 0.225755 | 0.020101 |
|  | FLC1 | 0.536232885 | 0.265674 | 0.025423 |
|  | APJ1 | 0.534168602 | 0.253274 | 0.02377 |
|  | BTT1 | 0.530739956 | 0.238978 | 0.021864 |
|  | EGD1 | 0.524433492 | 0.242966 | 0.022395 |
|  | PAC2 | 0.513915515 | 0.252968 | 0.023729 |
|  | CPR8 | 0.509940143 | 0.239072 | 0.021876 |
|  | FMO1 | 0.494968678 | 0.256062 | 0.024142 |
|  | CHS7 | 0.491054516 | 0.248479 | 0.023131 |
|  | CPR3 | 0.490928711 | 0.2301 | 0.02068 |
|  | FPR1 | 0.486417867 | 0.231394 | 0.020853 |
|  | PFD1 | 0.481465012 | 0.212909 | 0.018388 |
|  | EMC1 | 0.480338566 | 0.266601 | 0.025547 |
|  | PHB1 | 0.476629988 | 0.214656 | 0.018621 |
|  | OSM1 | 0.470548597 | 0.256366 | 0.024182 |
|  | CPR6 | 0.457176158 | 0.237478 | 0.021664 |
|  | SLP1 | 0.454189022 | 0.201533 | 0.016871 |
|  | EMC6 | 0.447335755 | 0.239155 | 0.021887 |
|  | SBA1 | 0.442336899 | 0.227983 | 0.020398 |
|  | GIM4 | 0.432131638 | 0.228682 | 0.020491 |
|  | PLP1 | 0.421151739 | 0.241901 | 0.022253 |
|  | CAJ1 | 0.374928859 | 0.238029 | 0.021737 |
|  | REJ5 | 0.373849089 | 0.247992 | 0.023066 |
|  | CIN4 | 0.358222333 | 0.218947 | 0.019193 |
| Unfolded Response | HSP42 | 1.625251874 | 0.126324 | 0.006843 |
|  | TCM62 | 1.410466482 | 0.149664 | 0.009955 |
|  | ULI1 | 1.151850745 | 0.151237 | 0.010165 |
|  | SSZ1 | 0.818483934 | 0.229362 | 0.020582 |
|  | NSG1 | 0.801033301 | 0.169288 | 0.012572 |
|  | MID2 | 0.711401431 | 0.162034 | 0.011605 |
|  | VMA22 | 0.691247529 | 0.246622 | 0.022883 |
|  | PMT1 | 0.668990056 | 0.081274 | 0.000836 |
|  | PTC2 | 0.634531422 | 0.132499 | 0.007667 |
|  | RBL2 | 0.624742611 | 0.165791 | 0.012106 |
|  | SPT20 | 0.599513718 | 0.121793 | 0.006239 |
|  | VPS74 | 0.598534481 | 0.135934 | 0.008125 |
|  | SSA2 | 0.589918406 | 0.107295 | 0.004306 |
|  | SSQ1 | 0.589008013 | 0.137328 | 0.00831 |
|  | DCR2 | 0.586887908 | 0.153281 | 0.010437 |
|  | ATP11 | 0.580284673 | 0.112514 | 0.005002 |
|  | TIM17 | 0.552027479 | 0.14591 | 0.009455 |
|  | VPS45 | 0.550980614 | 0.124994 | 0.006666 |
|  | EDE1 | 0.542110086 | 0.134086 | 0.007878 |
|  | YAR1 | 0.529598095 | 0.123981 | 0.006531 |
|  | ATP10 | 0.528314739 | 0.127588 | 0.007012 |
|  | SSA1 | 0.525491468 | 0.11805 | 0.00574 |
|  | SLT2 | 0.506206601 | 0.101307 | 0.003508 |
|  | LHS1 | 0.500114344 | 0.09811 | 0.003081 |
|  | COX20 | 0.485253712 | 0.123037 | 0.006405 |
|  | NSG2 | 0.476915463 | 0.124477 | 0.006597 |
|  | HAC1 | 0.465085389 | 0.126683 | 0.006891 |
|  | BXI1 | 0.461652469 | 0.141085 | 0.008811 |
|  | XDJ1 | 0.459205154 | 0.113851 | 0.00518 |
|  | YHI9 | 0.456087468 | 0.126035 | 0.006805 |
|  | PMT2 | 0.434734507 | 0.09461 | 0.002615 |
|  | PET100 | 0.430581802 | 0.112556 | 0.005007 |
|  | SHY1 | 0.422405668 | 0.125426 | 0.006723 |
|  | SSA4 | 0.400716528 | 0.11463 | 0.005284 |
|  | IRE1 | 0.386748703 | 0.122461 | 0.006328 |
|  | EPS1 | 0.384868348 | 0.117601 | 0.00568 |
|  | BCK1 | 0.382763973 | 0.097291 | 0.002972 |
|  | OPI1 | 0.365404158 | 0.116179 | 0.005491 |
| Translocation | YPT32 | 1.645631348 | 0.13659 | 0.008212 |
|  | SBH1 | 1.413830085 | 0.151529 | 0.010204 |
|  | PMR1 | 1.367843023 | 0.094089 | 0.002545 |
|  | SEM1 | 1.338697384 | 0.103644 | 0.003819 |
|  | SEC72 | 1.28015935 | 0.214548 | 0.018606 |
|  | SIL1 | 1.265714552 | 0.167225 | 0.012297 |
|  | SBH2 | 1.262056282 | 0.155554 | 0.010741 |
|  | RCY1 | 1.212762931 | 0.178603 | 0.013814 |
|  | SPO14 | 1.166218949 | 0.091477 | 0.002197 |
|  | SNC1 | 1.164148086 | 0.280509 | 0.027401 |
|  | MSO1 | 1.155457137 | 0.286611 | 0.028215 |
|  | SSH1 | 1.153373024 | 0.202384 | 0.016985 |
|  | SMY1 | 1.091140253 | 0.233384 | 0.021118 |
|  | PEP3 | 1.085329202 | 0.126096 | 0.006813 |
|  | SWF1 | 1.079273131 | 0.182869 | 0.014383 |
|  | SRO77 | 1.067431133 | 0.326433 | 0.033524 |
|  | SRO7 | 1.059426599 | 0.088412 | 0.001788 |
|  | VPS33 | 1.055348084 | 0.315666 | 0.032089 |
|  | OSH7 | 1.049267749 | 0.179742 | 0.013966 |
|  | KES1 | 1.048857383 | 0.377165 | 0.040289 |
|  | GYP5 | 1.03676655 | 0.387733 | 0.041698 |
|  | HES1 | 1.016854339 | 0.415389 | 0.045385 |
|  | MYO3 | 1.013012767 | 0.419455 | 0.045927 |
|  | SEC66 | 1.010455062 | 0.370787 | 0.039438 |
|  | OSH2 | 1.00204639 | 0.442515 | 0.049002 |
|  | MYO5 | 1.000652594 | 0.443329 | 0.049111 |
|  | SYT1 | 0.982140725 | 0.410492 | 0.044732 |
|  | SFH5 | 0.980243264 | 0.360606 | 0.038081 |
|  | SNC2 | 0.979664573 | 0.391737 | 0.042232 |
|  | MSB4 | 0.969225522 | 0.405469 | 0.044063 |
|  | OSH6 | 0.962424212 | 0.32229 | 0.032972 |
|  | TPM2 | 0.959841354 | 0.378071 | 0.040409 |
|  | OSH3 | 0.957332964 | 0.271499 | 0.0262 |
|  | TPM1 | 0.932703392 | 0.298355 | 0.029781 |
|  | MSB3 | 0.930291805 | 0.22828 | 0.020437 |
|  | YPT31 | 0.894456389 | 0.297473 | 0.029663 |
|  | KIN1 | 0.886896125 | 0.113166 | 0.005089 |
|  | GYL1 | 0.811371906 | 0.184856 | 0.014648 |
|  | SSA1 | 0.624327835 | 0.145127 | 0.00935 |
