# Supplementary figures and images for: Systematic Optimization of Protein Secretory Pathways in Saccharomyces cerevisiae to Increase Expression of Hepatitis B Small Antigen
Source: Front Microbiol. 2017 May 16;8:875. doi: 10.3389/fmicb.2017.00875 (PMC5432677; doi:10.3389/fmicb.2017.00875)

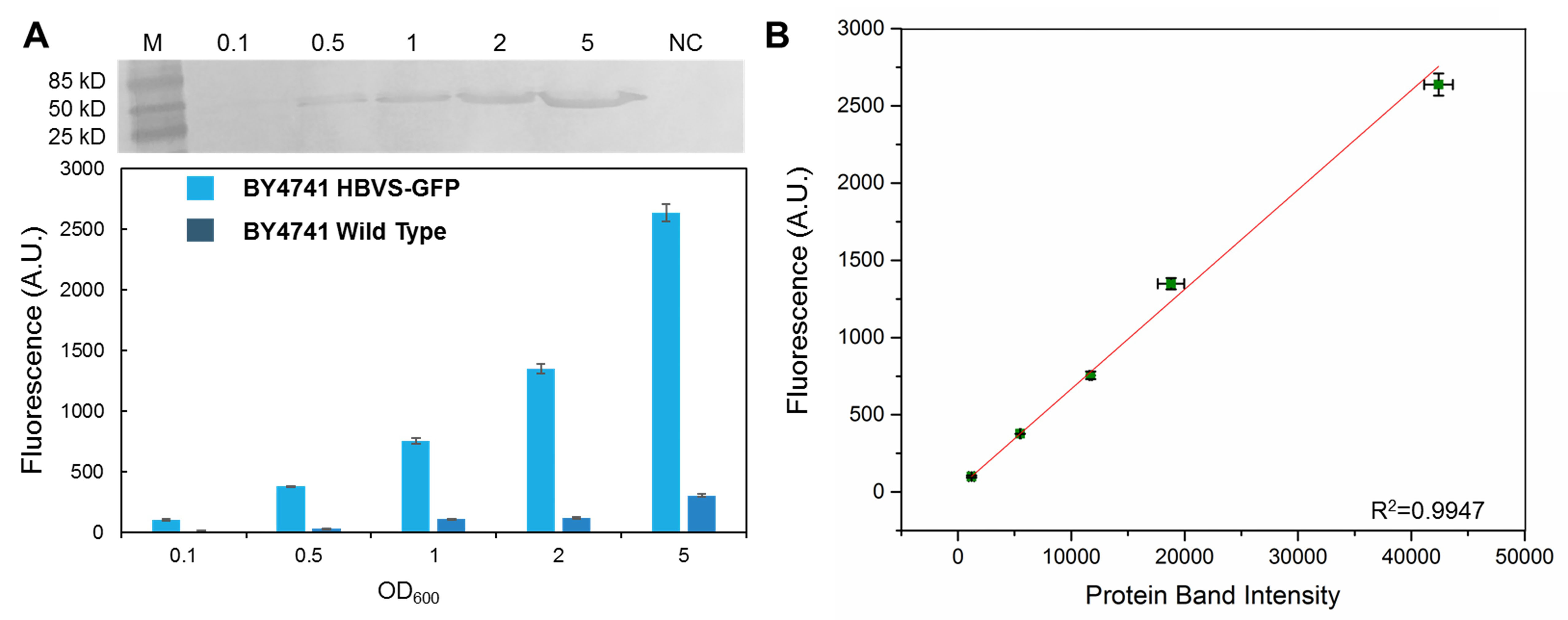

Supplement: Figure S1 — The correlation of the HBsAg-GFP protein expression level and the GFP fluorescence read by plate reader. (A) The western blotting of HBsAg-GFP proteins form culture with different OD600 and the corresponding fluorescence read by plate reader. Western blotting was repeated for three times. M, marker; NC, negative control; BY4741 HBS-GFP: BY4741 strain harboring the HBsAg-GFP expression plasmid; BY4741 Wild Type: a wild-type BY4741 strain without the HBsAg-GFP expression plasmid, which was used as the control to calibrate the autofluorescence of yeast cells. (B) Linear correlation between the western blotting reading and the GFP fluorescence (R2 = 0.99). The autofluorescence of yeast cells, although existing, was found to be ignorable in the correlation experiment. The protein band intensity was calculated by Quantity One software (Bio-Rad, USA). [file Image1.JPEG]

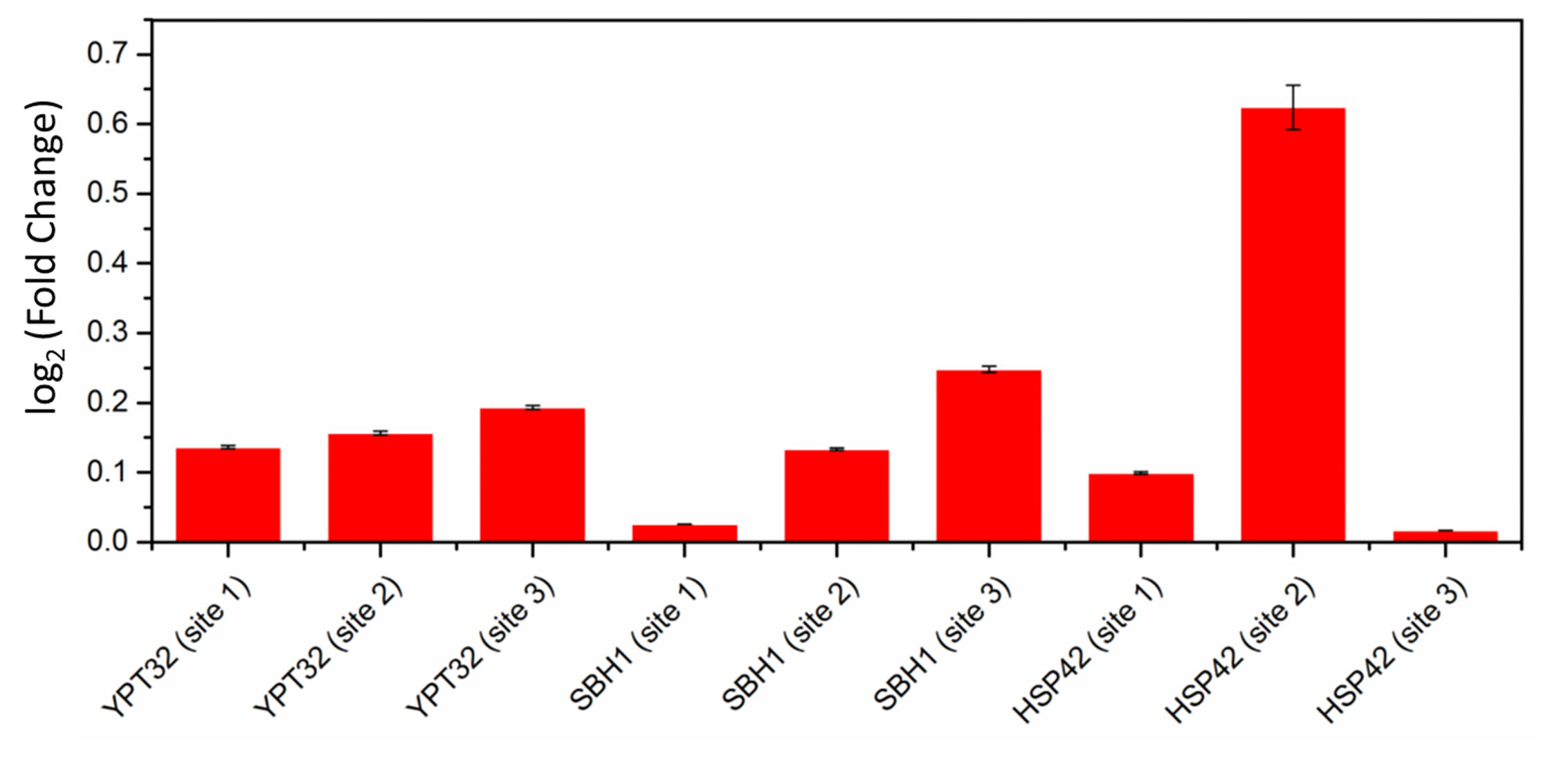

Supplement: Figure S2 — qPCR analysis of gene repression by CRISPRi system. The strains harboring the dCas9 plasmid and the plasmid containing individual gRNAs (e.g., YPT32 site 1) were grown in 25 mL SC media with all appropriate amino acids and 20 g/L glucose for 2 days. The strain harboring the dCas9 plasmid and an empty backbone plasmid for gRNAs was used as the control strain. To analyze gene expression of YPT32, SBH1, and HSP42, 1 ml of the cells were collected and the mRNA was extracted by yeast RNA extraction kit (Thermofisher, USA). Taqman primers designed for YPT32, SBH1, and HSP42 (Thermofisher, USA) were ordered from IDTDNA Inc. to amplify the target genes. ALG9 was used as the housekeeping gene during this assay. The Cq value (quantification cycle) for each sample was refactored into log2 (Fold Change) compared with the control strain. Each data point represented the mean of biological triplicates and the error bar was the standard deviation of log2 (Fold Change). [file Image2.JPEG]

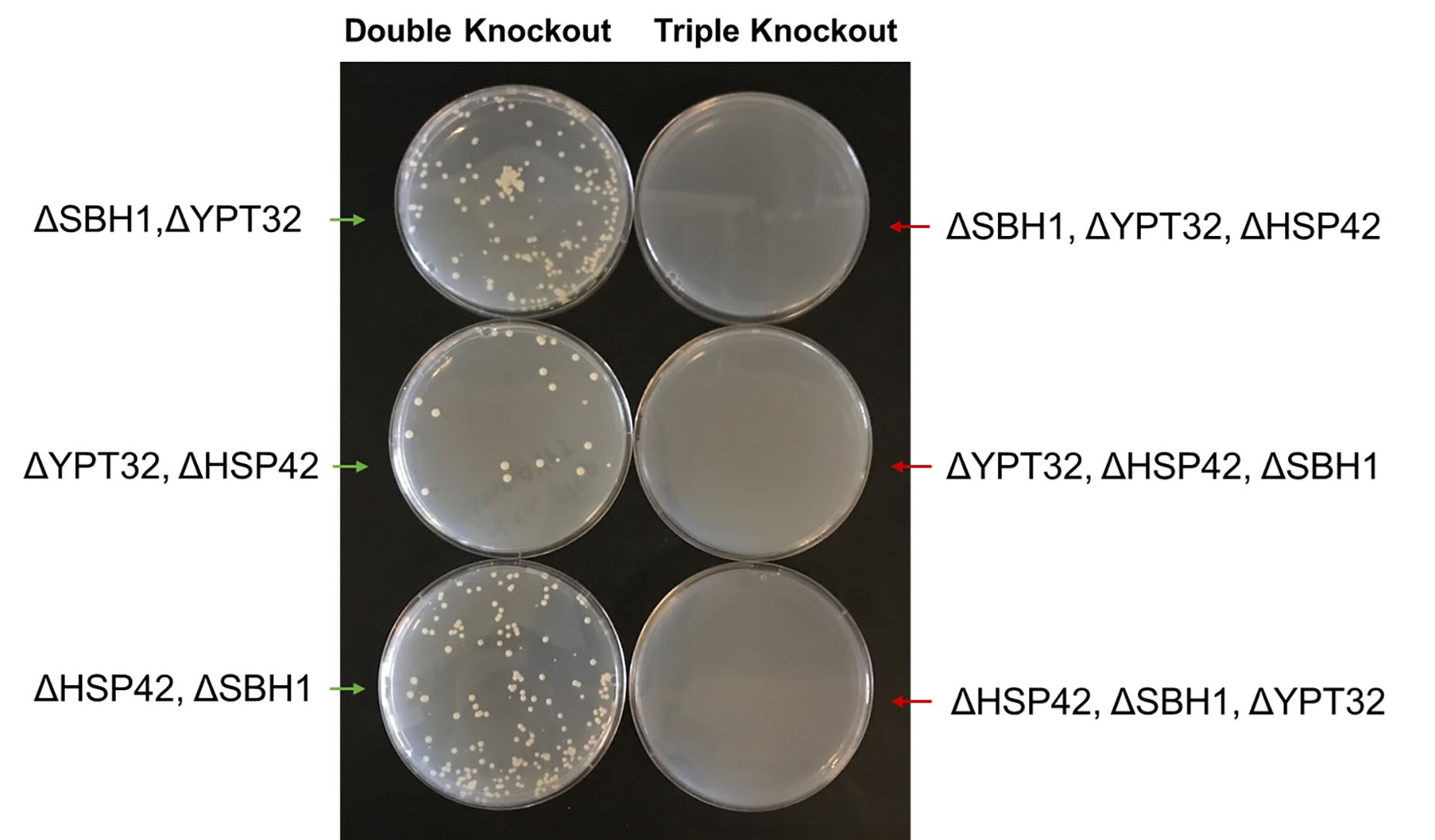

Supplement: Figure S3 — Construction of double- and triple- gene knockout strains. The protocol developed by Jay Keasling's group (Jakočiūnas et al., 2015) was used to construct the double- and triple- knockout strains. In brief, the wild-type Cas9 expression plasmid was purchased from Addgene (Plasmid #43804) and was transformed into three single knockout strains (i.e., ΔYPT32, ΔSBH1, and ΔHSP42) by using the lithium acetate transformation method. Appropriate gRNA plasmids (i.e., gRNAYPT32 to ΔSBH1 strain; gRNAHSP42 to ΔYTP32 strain and gRNASBH1 to ΔHSP42 strain) were used to guide the Cas9 protein to delete the target genes. One hundred nano grams of gRNA expression plasmid and 1 ng of 90-bp long dsOligos (introducing a stop codon during the DNA homologous repairing) were used per transformation. Yeast cells were then incubated on the galactose SC-medium plates for 3 days to generate the double-knockout strains: ΔSBH1ΔYPT32, ΔYPT32 ΔHSP42, ΔHSP42ΔSBH1. These double-knockout strains were then used as the parent strains to construct triple-knock strain: ΔSBH1ΔYPT32ΔHSP42. However, no colony was found for the triple-knock strain. [file Image3.JPEG]
